# Pterocarpan synthase (PTS) structures suggest a common quinone methide–stabilizing function in dirigent proteins and proteins with dirigent-like domains

**DOI:** 10.1074/jbc.RA120.012444

**Published:** 2020-06-21

**Authors:** Qingyan Meng, Syed G. A. Moinuddin, Sung-Jin Kim, Diana L. Bedgar, Michael A. Costa, Dennis G. Thomas, Robert P. Young, Clyde A. Smith, John R. Cort, Laurence B. Davin, Norman G. Lewis

**Affiliations:** 1 Institute of Biological Chemistry, Washington State University, Pullman, Washington, USA; 2 Earth and Biological Sciences Directorate, Pacific Northwest National Laboratory, Richland, Washington, USA; 3 Stanford Synchrotron Radiation Lightsource, Stanford University, Menlo Park, California, USA

**Keywords:** crystallography, dimerization, docking, plant biochemistry, plant defense, dirigent protein, lignans, lignins, pterocarpans, quinone methides

## Abstract

The biochemical activities of dirigent proteins (DPs) give rise to distinct complex classes of plant phenolics. DPs apparently began to emerge during the aquatic-to-land transition, with phylogenetic analyses revealing the presence of numerous DP subfamilies in the plant kingdom. The vast majority (>95%) of DPs in these large multigene families still await discovery of their biochemical functions. Here, we elucidated the 3D structures of two pterocarpan-forming proteins with dirigent-like domains. Both proteins stereospecifically convert distinct diastereomeric chiral isoflavonoid precursors to the chiral pterocarpans, (–)- and (+)-medicarpin, respectively. Their 3D structures enabled comparisons with stereoselective lignan– and aromatic terpenoid–forming DP orthologs. Each protein provides entry into diverse plant natural products classes, and our experiments suggest a common biochemical mechanism in binding and stabilizing distinct plant phenol–derived mono- and bis-quinone methide intermediates during different C–C and C–O bond–forming processes. These observations provide key insights into both their appearance and functional diversification of DPs during land plant evolution/adaptation. The proposed biochemical mechanisms based on our findings provide important clues to how additional physiological roles for DPs and proteins harboring dirigent-like domains can now be rationally and systematically identified.

Dirigent protein (DP) (Latin: *dirigere*, to guide or align) (1) biochemical functions give entry into distinct complex plant phenol metabolic classes. DPs apparently began to functionally emerge during evolutionary transition of “primitive” aquatic plants to land. Phylogenetic analyses have indicated the presence of numerous subfamilies (*i.e.* DIR-a to DIR-h (2, 3)) thus far (Fig. 1) throughout the plant kingdom.

**Figure 1.**
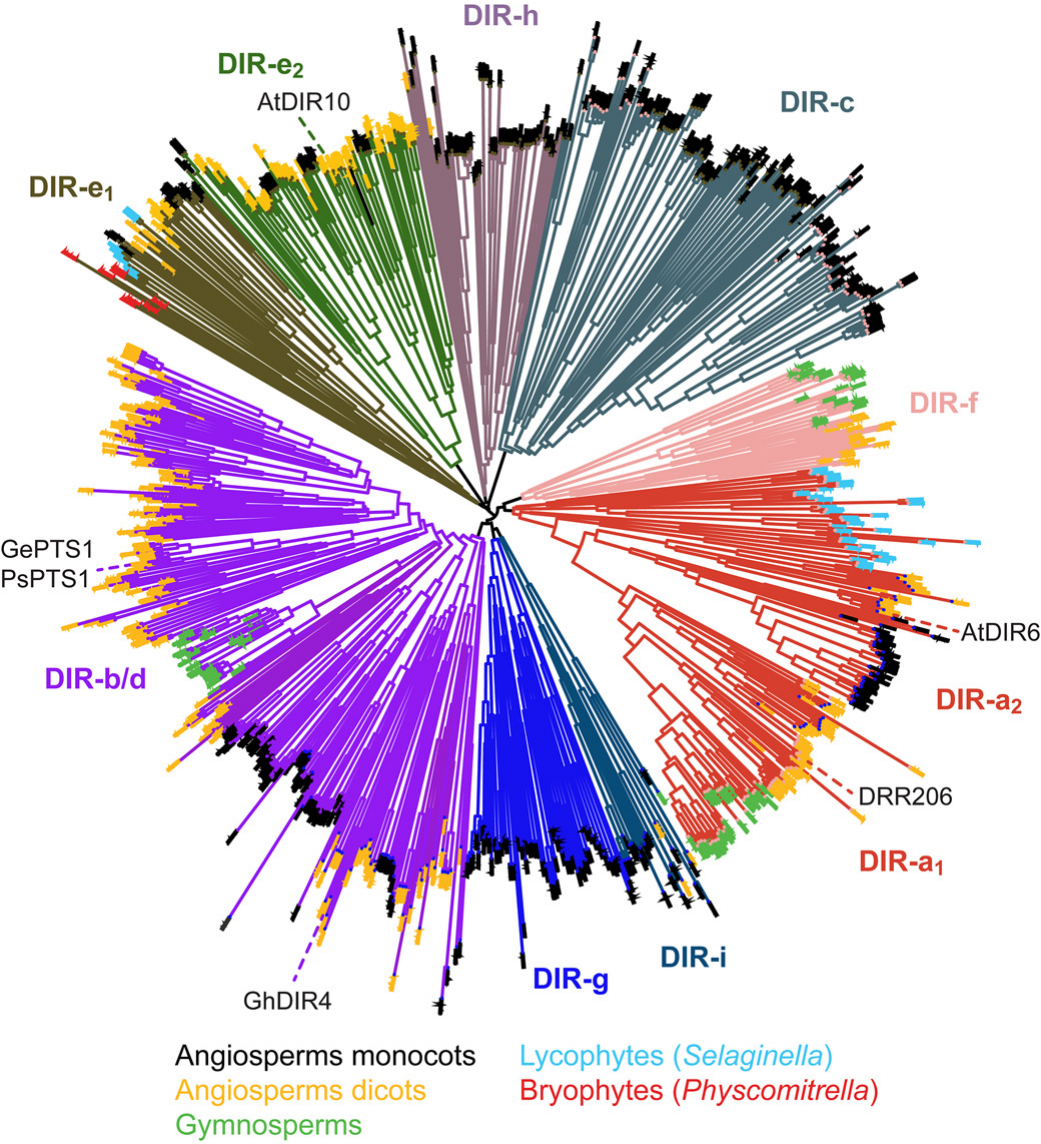
**Unrooted phylogenetic tree of dirigent and dirigent-like family proteins (Pfam PF03018).** The subfamily nomenclature of Ralph *et al*. (2) is maintained, with some families split where clear divisions were apparent (*e.g.* DIR-a_1_ and DIR-a_2_). Proteins whose functional characterization has been described in the literature are indicated (*e.g.* DRR206 (12, 14), a (+)-pinoresinol–forming DP from pea (*P. sativum*), in the Dir-a_1_ subfamily; AtDIR6 (8, 9, 13), a (–)-pinoresinol–forming DP from *A. thaliana*, in the Dir-a_2_ subfamily; GePTS1 (19) and PsPTS1, medicarpin-forming DPs from licorice (*G. echinata*) and pea, respectively, in the Dir-b/d subfamily; GhDIR4 (16, 17), an aromatic diterpenoid ((+)-gossypol–forming) DP from cotton (*G. hirsutum*); and AtDIR10 (20), a Casparian band lignin-forming DP from *A. thaliana*, in the Dir-e subfamily). The narrow distributions of sequences from gymnosperms, lycophytes, and bryophytes are easily discernable and contrast with the broad distribution of angiosperm dicots and even broader distribution of extant angiosperm monocots (mainly crop grasses). Ends of each branch of the tree are *colored* for different land plant families as indicated (*e.g. light blue ends* indicate lycophytes).

DP multigene families currently span liverworts (*e.g. Marchantia polymorpha*) (4), mosses (*e.g. Physcomitrella patens* (5) and *Sphagnum phallax* (RRID:SCR_006507)), lycophytes (*e.g. Selaginella moellendorffii* (6)), gymnosperms (*e.g. Picea* sp., (2) and *Thuja plicata* (7)), and angiosperms (*e.g. Arabidopsis thaliana* (2, 7–9) and *Linum usitatissimum*) (3, 10) (Fig. 1); DPs are absent in algae and cyanobacteria (3). However, most DPs (>95%) have no known biochemical function. All DPs and proteins harboring dirigent-like domains can be conveniently classified according to whether they contain the Pfam PF03018 domain (3, 11). To date, all DP subfamilies with known biochemical roles have been demonstrated to utilize different plant phenol substrates to gain entry into distinct plant phenol skeletal metabolic classes.

## Lignan-forming DPs

**Figure 2.**
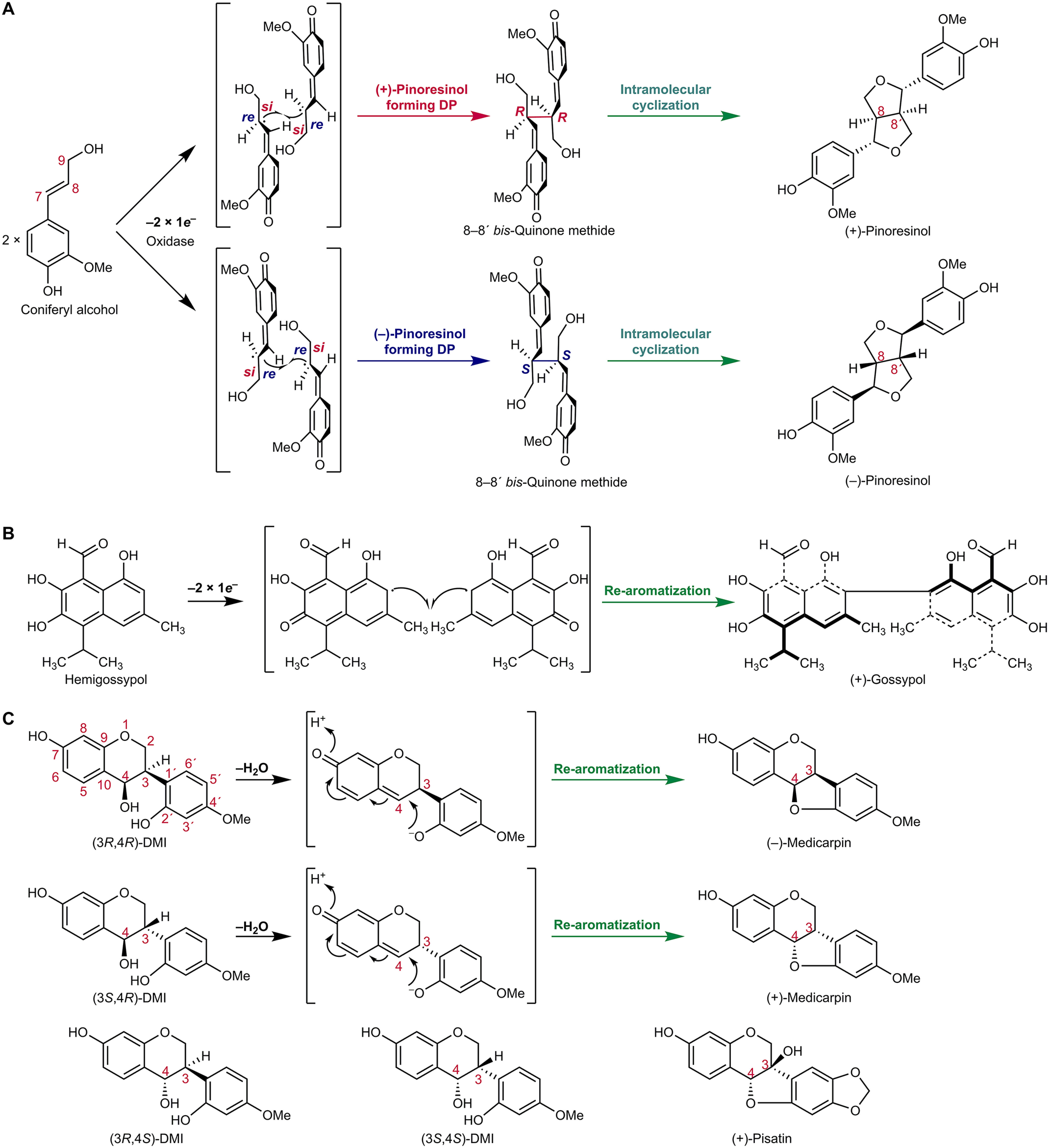
**Proposed general biochemical mechanism of DPs involving generation and stabilization of mono- or bis-quinone methides.** *A*, formation of either (+)- or (–)-pinoresinol from achiral coniferyl alcohol. Initially, 1e^–^ oxidation generates an intermediary prochiral free radical mono-quinone methide, which undergoes either *si*-*si* or *re*-*re* coupling to afford the chiral 8–8′-bis-quinone methides, depending on the pinoresinol-forming DP, to give after intramolecular cyclization either (+)- or (–)-pinoresinol, respectively. *B*, (+)-gossypol–forming DP whose action requires 1e^–^ oxidation of achiral hemigossypol to afford the proposed prochiral free radical mono-quinone methide intermediate. Stereoselective coupling then gives the bis-quinone methide derivative, re-aromatization of which generates (+)-gossypol. *C*, medicarpin-forming DP using chiral isoflavonoid substrates (3*R*,4*R*)-DMI, and (3*S*,4*R*)-DMI. The proposed biochemical mechanism involves mono-quinone methide generation and intramolecular cyclization/re-aromatization. (3*R*,4*S*)-DMI and (3*S*,4*S*)-DMI are poorer substrates. (+)-Pisatin is another example of a pterocarpan.

The first DPs reported were the (+)- and (–)-pinoresinol–forming DPs affording entry into the lignan metabolic pathways (*i.e.* provided that one-electron (1e^–^) oxidation capacity was also present) (1, 7–10, 12–15) (Fig. 2*A*). In this way, the (+)- and (–)-pinoresinol-forming DPs (DIR-a subfamily members, Fig. 1) engender distinct stereoselective intermolecular couplings, in the presence of a 1e^–^ oxidase or oxidant, of the prochiral coniferyl alcohol quinone methide (QM) free radicals so formed (*i.e.* to give the two distinct enantiomeric forms of pinoresinol, depending upon the Dir-a subfamily DP type). Conversely, in the absence of the DPs, only nonregiospecific and nonstereoselective phenoxy radical coupling occurs to afford a mixture of racemic products.

(+)-Pinoresinol– or (–)-pinoresinol–forming DPs initially afford formation of enantiomeric bis-QM intermediates, via either *si-si* or *re-re* coupling, depending upon the DP in a particular plant species (see Fig. 2*A*). Following this C–C bond formation, these bis-QM intermediates can then undergo intramolecular cyclization (C–O bond formation) to give the lignans (+)- or (–)-pinoresinols, respectively (Fig. 2*A*).

The (+)- and (–)-pinoresinol–forming DPs in subfamily DIR-a have been reported in a variety of plant systems, such as *Forsythia intermedia* (1), *Podophyllum peltatum* (15), western red cedar (*T. plicata*) (7), *A. thaliana* (8, 9, 13), flax (*L. usitatissimum*) (10), and pea (*Pisum sativum*) (12, 14). Pinoresinol is the biosynthetic entry point to many 8–8′-linked bioactive lignans, including several that have important roles in protecting against onset of different cancers and/or in clinically treating cancers (8).

## Aromatic diterpenoid-forming DPs

In a somewhat analogous manner, in aromatic terpenoid biosynthesis (16), the (+)-gossypol–forming DP, GhDIR4 (17), in the DIR-b/d subfamily, helps engender stereoselective intermolecular coupling (C–C bond formation) of achiral hemigossypol moieties, provided there is an 1e^–^ oxidase or oxidant. Again, in the absence of the DP, only racemic gossypol is formed.

Stereoselective coupling, however, affords formation of the presumed chiral bis-QM, re-aromatization of which gives entry into the aromatic diterpenoid class, in this case (+)-gossypol (Fig. 2*B*). Gossypol occurs in leaves, roots, and seeds of cotton (*Gossypium hirsutum*) and imparts resistance against herbivorous insects and pathogens, but (–)-gossypol is toxic to animals. The ratio of (+)- to (–)-gossypol in cottons grown in the United Statesis ∼3:2, although it can be as high as 98:2 in moco cotton, such as in the variety *marie-galante* (16).

## Pterocarpan-forming DPs

In pterocarpan (phytoalexin) biosynthesis studies, such as to (+)-pisatin in pea (Fig. 2*C*), it was deduced that DPs in the DIR-b/d subfamily were involved (18).^4^ Based on this deduction, Dr. Tomoyoshi Akashi, following completion of his term as a visiting scientist in the research group of the late Hans Van Etten, examined formation of the structurally related (–)-medicarpin in licorice (*Glycyrrhiza echinata*) on returning to Japan.

This led to the report of a medicarpin-forming DP (GePTS1) in the DIR-b/d family able to convert (3*R*,4*R*)-7,2′-dihydroxy-4′-methoxyisoflavanol (DMI) and (3*S*,4*R*)-DMI into (–)- and (+)-medicarpins, respectively, via lost of water and intramolecular C–O bond formation (19) (Fig. 2*C*). From its amino acid sequence, GePTS1 is a protein harboring dirigent-like domains.

## Lignin-forming DPs

In addition to the DPs in the above diverse metabolic pathways, cell wall structural reinforcement via lignin deposition has been implicated to involve DIR-e subfamily members (*e.g. Arabidopsis* AtDIR10; Fig. 1) (20, 21) in the angiosperms at least. The latter DPs are reportedly part of supramolecular complexes in enabling another metabolic product, lignin, to be formed in Casparian band tissues. However, the actual physiological substrates that these DPs utilize have neither been identified nor demonstrated *in vitro*.

The genes encoding DPs for entry points in pterocarpan, lignan, lignin biopolymer, and aromatic terpenoid biosynthesis are all of similar size. Of these DPs, the DIR-e lignin-forming DPs have much longer β1-β2 loops in their 3D structures, when compared with other DP's (*e.g.* DRR206 (12), AtDIR6 (22), and GhDIR4 (17). However, the biochemical significance of these much longer β1-β2 loops is currently unknown.

Many DP sequences contain canonical *N*-linked glycosylation motifs, and some have been confirmed experimentally as being post-translationally glycosylated, such as FiDIR1 (23), DRR206 (12, 14), AtDIR6 (9, 22), and GhDIR4 (17). On the other hand, the medicarpin-forming DP appears to have no requirement for post-translational glycosylation.

With the availability of structures of stereoselective medicarpin-forming DPs (nonglycosylated), stereoselective lignan-forming DPs (both apparently requiring post-translational glycosylation for stability), and a homology-modeled aromatic diterpenoid DP (GhDIR4), it was instructive to probe and compare the mechanistic biochemical features of these distinct DP types.

Described herein are the 3D structures of two stereoselective pterocarpan-forming DPs from pea and licorice, which preferentially produce either (+)- or (–)-medicarpin, depending on the substrate (Fig. 2*C*). These findings are discussed in the context of this DP type, which has dirigent-like (amino acid sequence similarity) domains as compared with the stereoselective lignan and aromatic terpenoid-forming DPs. Of particular interest was whether there was a common DP biochemical mechanism and, if so, what were the underlying mechanistic principles involved.

We describe that pterocarpan synthases, containing dirigent-like domains, initially engender mono-QM formation from their chiral substrates, this being followed by intramolecular cyclization (C–O bond formation) to afford entry into the pterocarpan natural product (phytoalexin) class. These differ from the other dirigent protein types, which instead initially enable stereoselective, one-electron, intermolecular coupling (C–C bond formation) of two identical achiral aromatic precursors to give chiral bis-QMs. The latter then either undergo intramolecular cyclization (C–O bond formation) or re-aromatization, respectively, to generate lignan and aromatic diterpenoid natural product classes.

## Results

**Figure 3.**
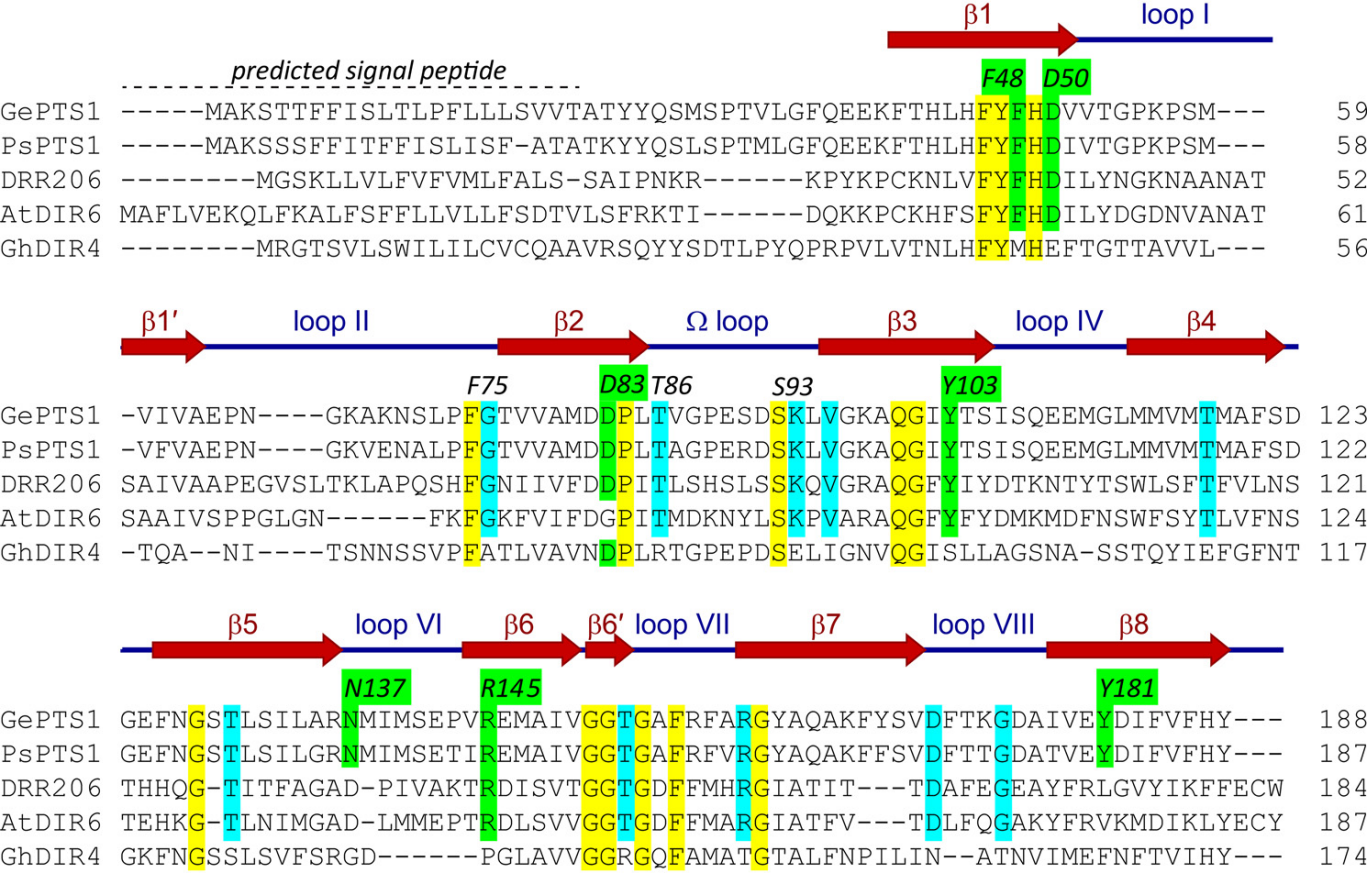
**Structure-based sequence alignment.** The structures of GePTS1 and PsPTS1, together with earlier structures for DRR206 and AtDIR6, help resolve ambiguities in the alignment, particularly in the last strand (β8). β-Strands in GePTS1 are shown with a *dark red arrow*, whereas a *blue line* indicates the loops in between the β-strands. Amino acid numbering (e.g. *F48* and *D50*) is that of GePTS1. *GePTS1*, *G. echinata* pterocarpan synthase 1; *PsPTS1*, *P. sativum* pterocarpan synthase 1; *DRR206*, *P. sativum* (+)-pinoresinol–forming DP; *AtDIR6*, *A. thaliana* (–)-pinoresinol–forming DP; *GhDIR4*, *G. hirsutum* (+)-gossypol–forming DP.

The *P. sativum* “Cam_eor” Unigene set (24) was searched using *GePTS1* as query, which resulted in a gene (PsCam039127) being selected as possibly encoding a medicarpin-forming DP. Trivially named *PsPTS1,* it has ∼92%/85% sequence similarity/identity to GePTS1 at the amino acid level. Fig. 3 shows amino acid sequence alignments of the medicarpin-forming DPs (GePTS1 and PsPTS1), the (+)- and (–)-pinoresinol–forming DPs (DRR206 and AtDIR6) from *P. sativum* and *A. thaliana*, respectively, and the aromatic diterpenoid (+)-gossypol–forming DP (GhDIR4) in *G. hirsutum*.

### Heterologous expression and gel-permeation chromatography

*GePTS1* and *PsPTS1* coding sequences were individually codon-optimized for *Escherichia coli*, with each synthetic gene cloned into the pET101/D-TOPO^®^ *E. coli* expression vector harboring a C-terminal 6× polyhistidine region. The vector constructs were then each used to transform *E. coli* BL21 (DE3) cells. After induction with isopropyl 1-thio-β-d-galactopyranoside, the resulting recombinant His-tagged proteins were individually purified to apparent homogeneity (Fig. S1*A*) by use of metal-chelating affinity chromatography.

Gel-permeation chromatography (GPC) was next carried out on a TSKgel G3000SW_XL_ column, precalibrated with molecular weight standards, to determine the oligomeric state of both PTSs. GePTS1 and PsPTS1, in solution, exist mainly as trimers (∼68.0 kDa), with (because of association/aggregation) a small amount of higher-molecular weight entities also being evident (roughly corresponding to 410–500 kDa).

### DP assays

Next, both GePTS1 and PsPTS1 DPs were used in assays with racemic mixtures of the diastereomers obtained through chemical synthesis from racemic vestitone (see “Experimental procedures”) (*i.e.* either *cis*-DMI ((3*R*,4*R*) and (3*S*,4*S*)) or *trans*-DMI ((3*S*,4*R*) and (3*R*,4*S*)), respectively), with substrates and products easily resolved by chiral column chromatography (Chiral OJ column, Chiral Technologies). As reported previously (19), GePTS1 converted either (3*R*,4*R*)-DMI or (3*S*,4*R*)-DMI into (–)- or (+)-medicarpin, respectively (Fig. S2, *B* and *I*). The pea medicarpin-forming DP (PsPTS1) catalyzed the same conversions (Fig. S2, *C* and *J*). Control assays (no DP present) gave smaller amounts of racemic medicarpin products (Fig. S2, *A* and *H*) because of nonenzymatic conversion of *cis-* and *trans*-DMI.

Kinetic data for both DPs were next obtained as follows: assays were carried out in triplicate at 10 concentrations of *cis*-DMI ((3*R*,4*R*) and (3*S*,4*S*)) and *trans*-DMI ((3*S*,4*R*) and (3*R*,4*S*)) for 5 min. Triplicate assays were also carried out in the absence of DPs to account for the nonenzymatic conversion of *cis-* and *trans*-DMI. From these determinations, GePTS1 preferentially utilized the *cis*-DMI (3*R*,4*R*) isomer, whereas the (3*S,*4*S*) *cis*-DMI was not converted initially under these conditions (Table 1). In a somewhat analogous manner, the corresponding *trans*-DMI (3*S*,4*R*) isomer was utilized over the (3*R*,4*S*) *trans*-DMI form. Thus, kinetic data reported in Table 1 are calculated based on conversion of the *cis*-DMI (3*R*,4*R*) and *trans*-DMI (3*S*,4*R*) isomers. Moreover, these data established an ∼18-fold higher catalytic turnover, *k*_cat_/*K_m_*, for the (3*R*,4*R*) *cis*-DMI *versus* the corresponding (3*S*,4*R*) *trans*-DMI isomer.

**Table 1:** **Kinetic data for GePTS1, PsPTS1, and GePTS1 mutants*^a^***

	*cis*-DMI ((3*R*,4*R*) and (3*S*,4*S*))				*trans*-DMI ((3*S*,4*R*) and (3*R*,4*S*))			
	*K_m_*	*V*_max_	*k*_cat_	*k*_cat_/*K_m_*	*K_m_*	*V*_max_	*k*_cat_	*k*_cat_/*K_m_*
	μ*m*	*picokatals*/μ*g protein*	*s^–1^*	*m^–1^ s^–1^*	μ*m*	*picokatals*/μ*g protein*	*s^–1^*	*m^–1^ s^–1^*
GePTS1	145	2,674	59.9	412,800	680	712	15.9	23,440
PsPTS1	890	2,285	50.5	56,750	830	147	3.3	3,930
D50A	1,175	220	4.9	4,180	520	9	0.2	370
D83A	1,300	283	6.3	4,870	665	58	1.3	1,960
Y103F	555	306	6.9	12,340	3,320	265	5.9	1,830
Y181F	825	233	5.2	6,310	5,665	180	4.0	710

*^a^* under the assay conditions, *cis*-DMI (3*R*,4*R*) and *trans*-DMI (3*S*,4*R*) were only converted into products.

For PsPTS1, the assays to obtain kinetic parameters were exactly as described above. Again, the *cis*-DMI (3*R*,4*R*) isomer was utilized under these conditions, whereas the (3*S,*4*S*) *cis*-DMI was not. In our hands, PsPTS1 displayed much lower (∼7-fold) catalytic turnover (*k*_cat_/*K_m_*) relative to GePTS1, this in part being due to the ∼6*-*fold increase in *K_m_*.

Under these conditions, the corresponding *trans*-DMI (3*S*,4*R*) isomer was also utilized, whereas the (3*R*,4*S*) *trans*-DMI was not converted. However, the catalytic turnover (*k*_cat_/*K_m_*) was reduced ∼6-fold, relative to GePTS1, whereas the *K_m_* values were very similar for both PsPTS1 and GePTS1.

Additionally, the (3*S*,4*S*) and (3*R*,4*S*) enantiomers were slowly converted into (+)- and (–)-medicarpin when >1 μg of DP was used in the assays and when longer incubation times were used (30 min or more; data not shown).

### Medicarpin-forming DP structure determinations

Medicarpin-forming DP (GePTS1 and PsPTS1) crystals were obtained as described under “Experimental procedures” following initial screening at the Hauptman Woodward Institute (Buffalo, NY), where 1,536 conditions were tested (25).

**Figure 4.**
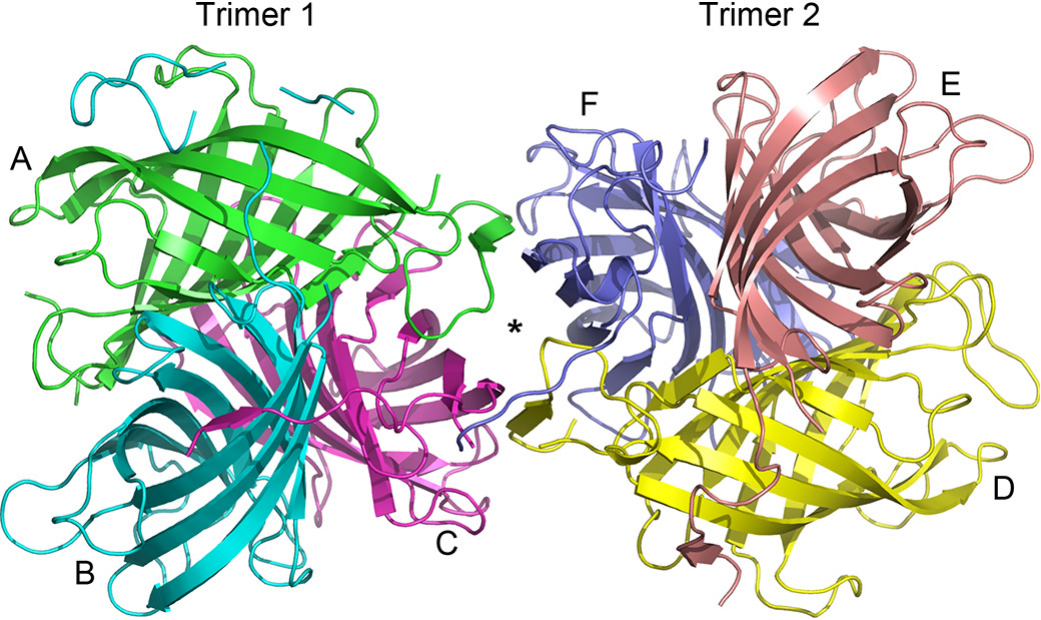
***Ribbon representation* of the GePTS1 structure.** The biological unit is a tightly packed trimer, and the asymmetric unit contains a dimer of trimers related by a pseudo-2-fold axis perpendicular to the plane of the paper. Trimer 1 on the *left* comprises monomers A (*green*), B (*cyan*), and C (*magenta*), and trimer 2 comprises monomers D (*yellow*), E (*pink*), and F (*blue*) on the *right*.

The GePTS1 structure was solved by molecular replacement at 2.6 Å resolution (Fig. S3*A*). Six independent DP monomer molecules, labeled *A–F* (Fig. 4), were located in the crystallographic asymmetric unit, arranged as a dimer of trimers. The buried surface area between the two trimers is small, and the biologically active form in solution is presumed to be a trimer, as confirmed by GPC analysis.

In any event, the two trimers from the X-ray analysis are related by a noncrystallographic symmetry 2-fold axis roughly parallel to the body diagonal of the P3_1_21 asymmetric unit. The residues in each of the six GePTS1 monomers are as follows: A, 23-65, 72-189; B, 26-29, 38-194; C, 26-189; D, 23-190; E, 24-197; F, 24-191 (Fig. 3). The mature sequence of GePTS1 begins at residue Ala^23^ and ends at Tyr^188^. Additional electron density was observed extending away from the C terminus to various extents in all six monomers. Monomers A and C have one additional residue that could be modeled, monomer D has two residues, and monomer F has three. Nine additional residues were modeled in monomer E, and monomer B has an additional 19 residues. The latter were identified as part of the linker for the C-terminal His tag from the pET101/D-TOPO^®^ expression vector (Fig. S4).

**Figure 5.**
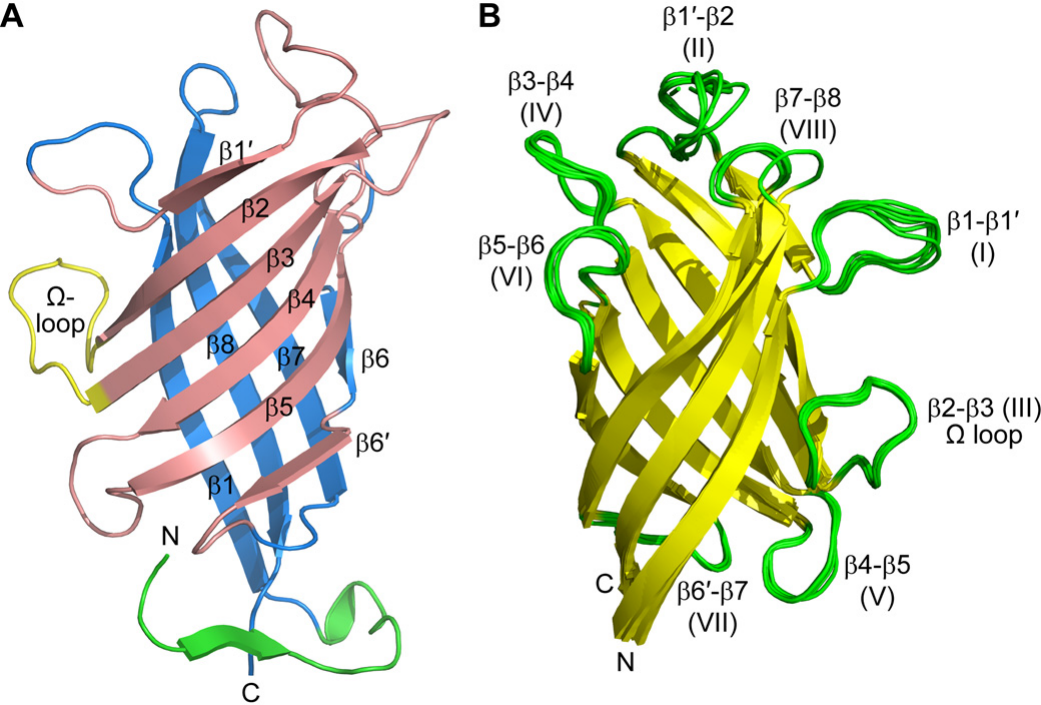
**Structure of the GePTS1 monomers.** *A*, *ribbon representation* of the eight-stranded barrel *colored* as two β-sheets in *pink* and *blue*. The N-terminal region is *colored green*. An Ω loop between strands β2 and β3 is *colored yellow*. The secondary structure labeling is also shown. *B*, superposition of the six monomers onto each other.

The GePTS1 monomer is an eight-stranded antiparallel β-barrel comprised of two curved anti-parallel sheets formed by strands β1′, β2, β3, β4, β5, and β6′ (designated sheet I) and β6, β7, β8, and β1 (designated sheet II) (Fig. 5*A*) that contact each other only slightly at the β1′-β8 and β5-β6′ interfaces. The N and C termini are located in adjacent β-strands at one end of the barrel. The six monomers superimpose onto each other with root mean square deviations (RMSDs) in Cα positions of between 0.53 and 1.05 Å. Inspection of the superimposed structures shows that the β-barrels align almost perfectly, with the main deviations occurring in the N and C termini and in loops at the opposite end of the molecule (Fig. 5*B*). If the three monomers comprising a trimer are superimposed, the N and C termini are quite divergent in structure, but when the molecules are related by the noncrystallographic symmetry 2-fold (A–D, B–E, and C–F), there is a much closer structural similarity. Further inspection of the two trimers as a whole show that the N- and C-terminal extensions wrap around neighboring monomers. The interface between the two trimers in the asymmetric unit is, however, not compact, and the protein is not likely to adopt the hexameric (dimer of trimers) state in dilute solution (*i.e.* as demonstrated with the GPC analyses above).

The pea medicarpin-forming DP, PsPTS1, structure was solved by single-wavelength anomalous diffraction methods using the signal from intrinsic sulfur atoms (sulfur-SAD (single anomalous diffraction)) in the 11 methionine residues (excluding the N-terminal methionine). Its structure was refined against 1.5 Å resolution native data to a final *R*_free_ of 0.1907 (Fig. S3*B*). It consists of a single monomer in the asymmetric unit, residues Phe^35^–Tyr^187^, plus two residues at the C terminus (Lys^188^ and Gly^189^) from the linker for the C-terminal His tag. The extended N terminus observed in some of the GePTS1 monomers is not resolved in the PsPTS1 structure. A trimeric complex is formed by the crystallographic 3-fold axis parallel to the body diagonal of the cubic unit cell, this being in agreement with the GPC. PsPTS1 also has an eight-stranded β-barrel structure (Fig. S5), comprised of two curved β-sheets with the same topology as GePTS1 (sheet I: β1′, β2, β3, β4, β5, and β6′; sheet II: β6, β7, β8, and β1).

The PsPTS1 and GePTS1 monomer structures are thus very similar, with RMSDs between 0.47 and 0.75 Å for the superposition of the PsPTS1 monomer on the six independent GePTS1 monomers. The entrance to the putative active site of the GePTS1 and PsPTS1 monomers is located at the end of the barrel opposite the N and C termini (Fig. 5*B* and Fig. S5). The opening of the active site is surrounded by five loops that show some degree of structural differences in the six monomers. These loops are between strands β1 and β1′ (loop I), strands β1′ and β2 (loop II), strands β3 and β4 (loop IV), strands β5 and β6 (loop VI), and strands β7 and β8 (loop VIII). The other loops, III (Ω), V, and VII, project out from the side of the monomer opposite that involved in trimer formation.

**Figure 6.**
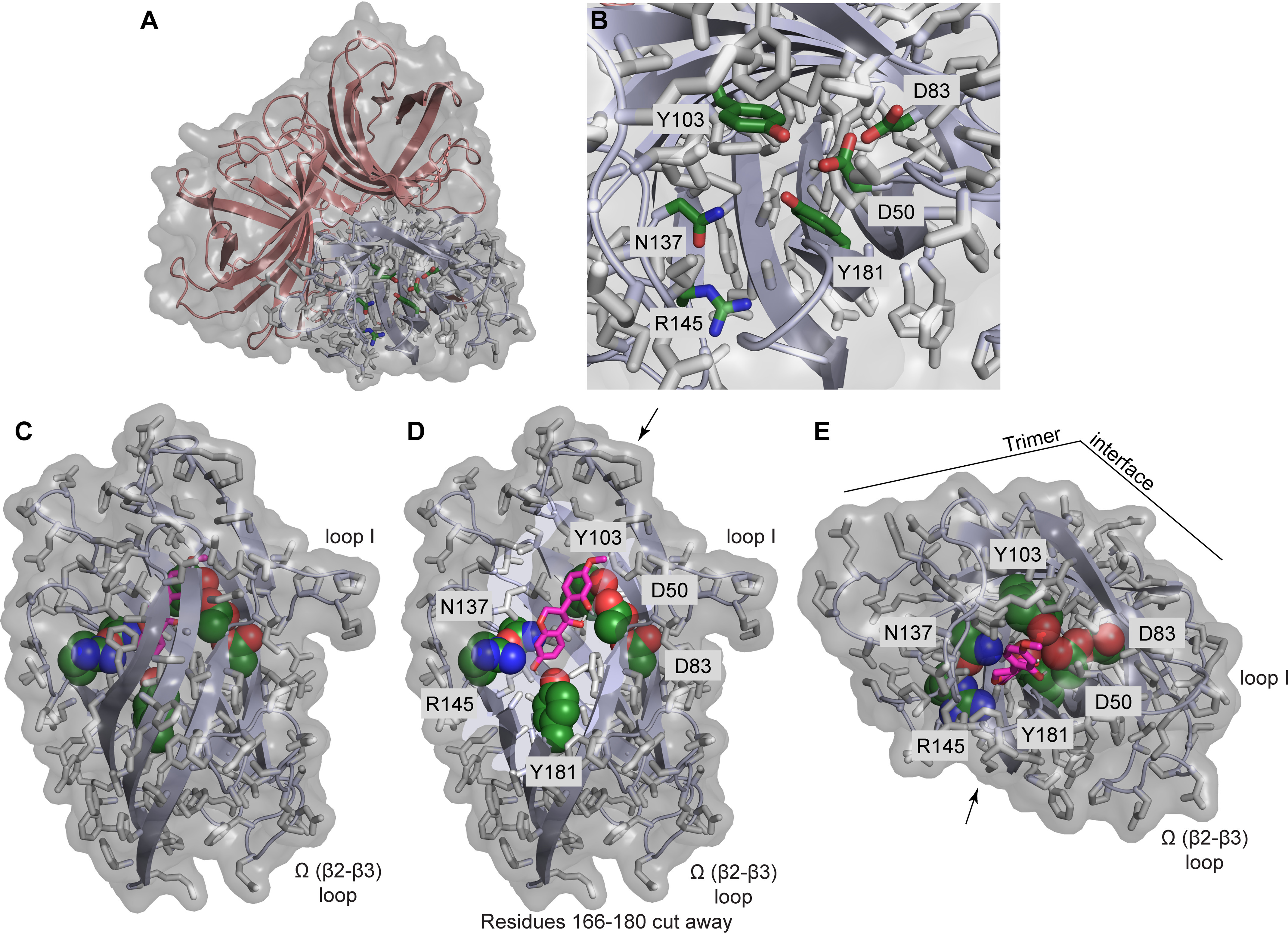
**The GePTS1 active site.** *A*, depiction of the GePTS1 trimer in *cartoon mode* with *transparent surface*, viewed from the *top* near the 3-fold symmetry axis, showing side-chains of polar active-site residues (Asp^50^, Asp^83^, Tyr^103^, Asn^137^, Arg^145^, and Tyr^181^) for one monomer as *sticks* with *dark green* carbon atoms. *B*, *zoom-in view* of the active site. *C*, *side view* of GePTS1 monomer with docked (*3S,4R*)-DMI substrate (*pink carbon atoms*) indicating the degree to which the substrate can be buried within the barrel interior. *D*, same side view with residues 166–180 cut away to reveal the active-site tunnel with docked (*3S,4R*)-DMI and the polar active-site residues. *E*, *top view* of GePTS1 monomer with docked (*3S,4R*)-DMI, looking directly into the active-site tunnel. *Arrows* in *D* and *E* indicate the viewer's perspective shown in the other *panel*, and key loops and the trimer interface are indicated as reference points. The PsPTS1 active site is essentially identical to that of GePTS1 in terms of the residues present and their side-chain rotamers.

The putative active-site cavity is a tunnel that extends into the barrel to a depth of ∼18 Å from the outermost external loops. For example, in GePTS1, the cavity volumes range between 350 and 500 Å^3^ (calculated with ICM-Pro (26)) and are lined by predominantly aromatic and hydrophobic residues and two aspartate residues (Asp^50^ and Asp^83^) (Fig. 6*B*). (The GePTS1 and PsPTS1 residues lining the interior pocket and forming the putative active site are identical, with all important/conserved residues numbered as in GePTS1 (Fig. 3) in the following discussion.) The roughly cylindrical active-site cavity is long and narrow (around 7 Å diameter) and nearly parallel to the 3-fold symmetry axis of the trimer, where Tyr^181^ sits at the base of the tunnel with its hydroxyl group projecting along the tunnel axis. Additionally, lining the tunnel are polar residues Asn^137^ and Arg^145^, conserved in both PTSs and in many DIRb/d sequences (Fig. S6). Compared with DRR206 and AtDIR6 (PDB ID 4REV and 5LAL, respectively), the GePTS1 and PsPTS1 active sites are narrower and deeper and aligned more parallel with the trimer symmetry axis, whereas DRR206 and AtDIR6 are wider and shallower and point outward more. Like DRR206 and AtDIR6, GePTS1 and PsPTS1 structures contain an Ω loop (Figs. 5 and 6*C* and Fig. S5) that folds back to contact the exterior of the barrel with conserved loop residues Thr^86^ and Ser^93^ (Fig. 3) forming a cluster with the highly conserved residue His^49^ on the barrel itself. A second exterior loop on the same side of the barrel occurs at the end of β1 prior to β1′ (Fig. 5*B* and Fig. S5). A similar loop and the following short β-strand are present in the structure of AtDIR6, but not in DRR206, where the corresponding sequence is disordered. Finally, a conserved β-bulge is found in both GePTS1 and PsPTS1 structures near the end of β7.

### Docking studies

Docking of DMI substrates (3*S*/*R*,4*R*-DMI) and the presumed 3*S*/*R*-DMI-QM intermediate in the GePTS1 active site (Fig. 6, *C–E*) showed that they could potentially bind in a lengthwise fashion in the active-site tunnel with either end pointing in toward the conserved Tyr^181^ residue at the base of the tunnel. These docking simulations were used to evaluate whether the proposed mechanism discussed below is plausible, given the constraints placed on it by the dimensions of the active site and the size of the substrate and intermediate, and to identify low-energy orientations of the bound substrates and intermediates that are consistent with this mechanism. Conserved polar residues Asp^50^, Asp^83^, Tyr^103^, Asn^137^, and Arg^145^ (Fig. 6*B*) are located along the sides of the tunnel, and the substrate and intermediate presumably must be able to bind in an orientation that places their key reactive components proximally to the necessary residues. These criteria were used to guide selection of the docked structures shown in Fig. 6 (*C–E*) for GePTS1. The preferred orientation thus has the QM oxygen at the bottom of the tunnel near Tyr^181^ and C-4 (bearing the labile OH in DMI) oriented toward Asp^50^. We found that for both DMI and DMI-QM, substrates with the *S* configuration at C-3 performed better in docking simulations. However, the substrate with the *R* configuration at C-3 appears to be the preferred substrate in plants. Furthermore, better docking was found when the pyran ring conformation was such that the phenol substituent on C-3 was equatorial, making the entire structure more flat and less bent, consistent with the straight and narrow nature of the active-site tunnel. We note that the simulations did not allow for any adjustment of side-chain conformations within the protein active site upon binding, which presumably could alter the specificity and ligand-binding interaction energy.

### GePTS1 mutants

Based on the above docking studies, four GePTS1 mutants were obtained (D50A, D83A, Y103F, and Y181F), these being synthesized using GeneOptimizer^®^ (Invitrogen). Each mutant was individually cloned into the *E. coli* pET100/D-TOPO^®^ expression vector. Following purification of each recombinant protein (Fig. S1*B*), the kinetic parameters were individually determined using either *cis*-DMI ((3*R*,4*R*) and (3*S*,4*S*)) or *trans*-DMI ((3*S*,4*R*) and (3*R*,4*S*)) substrates as above, with the kinetic data compared with that for WT GePTS1 (Table 1). Moreover, each of these mutants was subjected to CD analysis to verify proper folding (Fig. S7).

**Figure 7.**
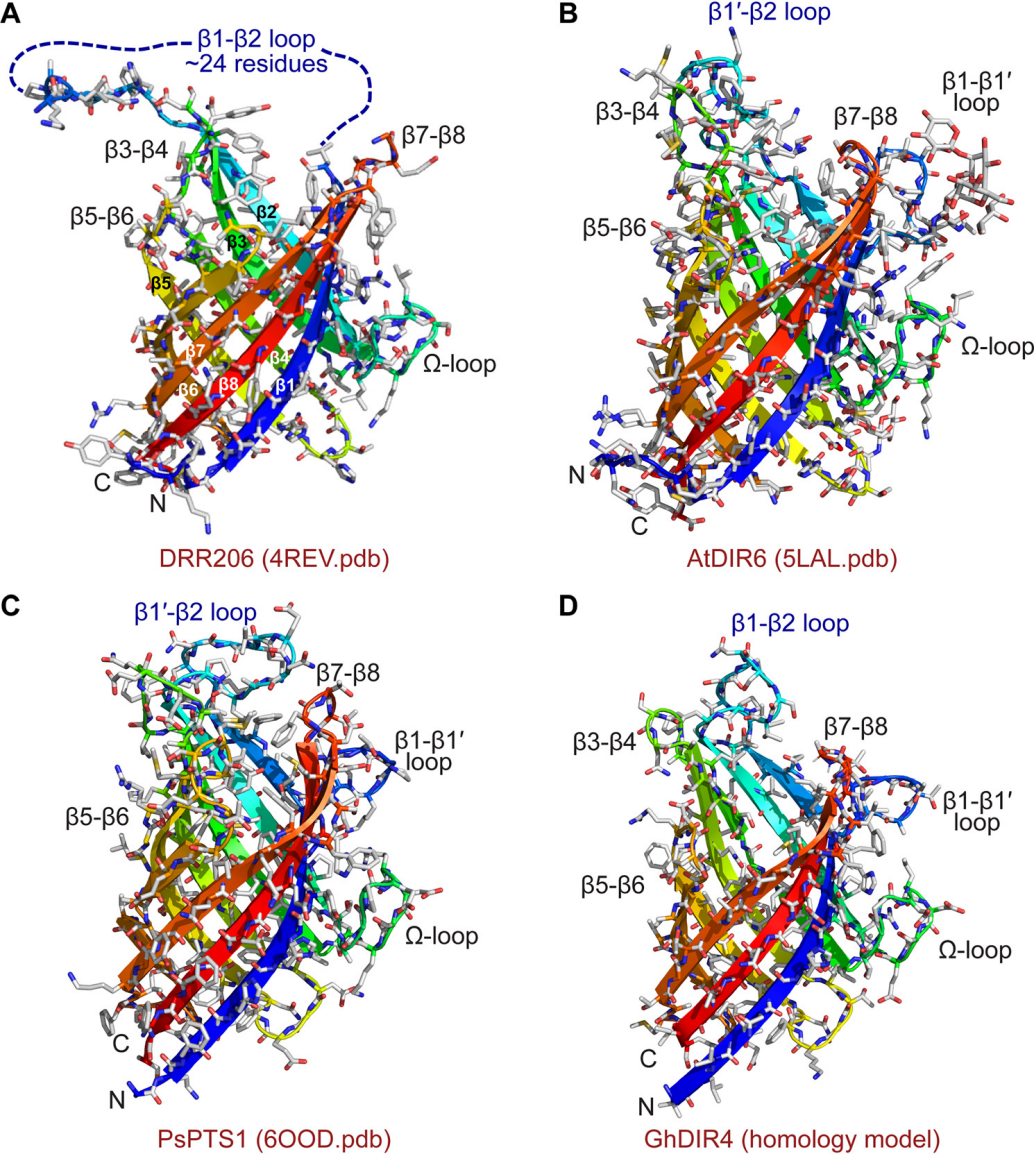
**3D structure and homology modeling of DPs.** Shown are 3D structures of DRR206 (4REV) (*A*), AtDIR6 (5LAL) (*B*), and PsPTS1 (6OOD) (*C*). *D*, homology model of GhDIR4 created with Phyre2 in one-to-one threading mode using PsPTS1 structure as a template (30, 31). The β-strands are *colored blue to red* from the N to the C terminus: *royal blue*, β-1; *slightly lighter blue*, β1′; *light blue-green*, β2; *green*, β3; *yellow-green*, β4; *yellow*, β5; *lighter orange*, β6 and β6′; *darker orange*, β7; *red*, β8.

All four GePTS1 mutants, when subjected to comprehensive kinetic analysis, displayed large reductions in catalytic turnover. In the mutant assays, only the *cis*-DMI (3*R*,4*R*) (Fig. S2, *D–G*) and *trans*-DMI (3*S*,4*R*) (Fig. S2, *K–N*) were again converted, albeit very poorly. In other words, using *cis*-DMI (3*R*,4*R*) as substrate, our kinetic data established that the D50A, D83A, Y103F, and Y181F mutants resulted in about 1.0, 1.2, 3.0, and 1.5% of the WT GePTS1 catalytic turnover. In a comparable manner, when the corresponding *trans*-DMI (3*S*,4*R*) isomer was utilized, catalytic turnover was 1.6, 8.3, 7.8, and 3.0% of that of WT GePTS1, respectively.

## Discussion

### Overall topology

The DRR206 (Fig. 7*A*) (12), AtDIR6 (Fig. 7*B*) (22), GePTS1 (Fig. 5*A*), and PsPTS1 (Fig. 7*C*) monomers, in their respective trimers, all have the same eight-stranded β-barrel topology. Of these, the (+)-pinoresinol–forming DP (DRR206) from pea, obtained at 1.95 Å resolution (12), was the first 3D DP structure (PDB entry 4REV) solved. Its structure contained two independent monomers in the asymmetric unit, and the trimeric structure was generated by the crystallographic 3-fold axis of the H3 space group. In the same way, the PsPTS1 trimer is also crystallographic, with the three monomers related by the 3-fold body diagonal of the cubic unit cell. The *Arabidopsis* (–)-pinoresinol–forming DP (AtDIR6) structure was also solved as two monomers, and the trimer was generated crystallographically (22).

Superposition of the PsPTS1 and GePTS1 monomers against DRR206 and AtDIR6 using secondary structure–matching algorithms (27) implemented in COOT (28) gave RMSDs ranging from 1.4 to 1.6 Å for the β-barrel core, slightly higher compared with those between the two PTS1 enzymes themselves (Table S1). The eight β-strands match very well between the PTS1 dirigent-like proteins and other two DPs, with the main differences occurring in the N and C termini and in the loops between the β-strands, in particular loops I, II, IV, and V. Interestingly, the Ω loop adopts the same conformation in all four enzymes, which hints at a functionality for this structural element as suggested for DRR206 (12). When the GePTS1 and symmetry-generated PsPTS1 trimers are superimposed upon the symmetry-generated DRR206 and AtDIR6 trimers, the core RMSDs are similar for the three β-barrel core, indicative of a highly conserved oligomeric structure. The RMSDs are significantly greater, however, when the Cα atoms of all residues are matched using ICM-Pro (26), primarily due to the conformational variability in the loop regions between these enzymes (Table S1).

Currently, the GePTS1 structure is the only one that shows the trimer without requiring it to be generated by crystallographic symmetry, being found as a dimer of trimers in the asymmetric unit. Furthermore, even though the gossypol-forming DP GhDIR4 (a member of the DIR-b/d family) has low sequence identity to GePTS1/PsPTS1 (∼35%) and AtDIR6/DRR206 (∼25%), these DP structure determinations allowed for homology modeling with reasonable quality for the core barrel structure (Fig. 7*D*) (29, 30).

### Ω loops and other alignments

**Figure 8.**
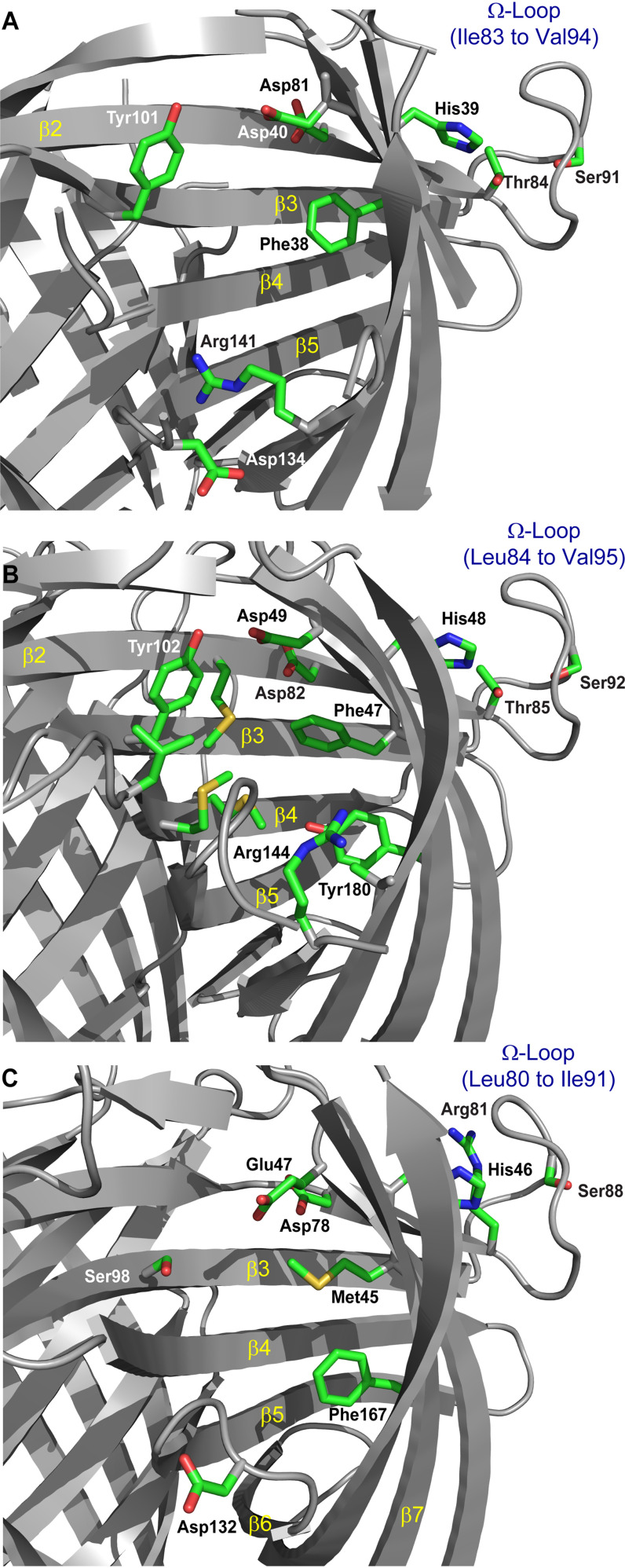
**Putative DP active-site pockets.** *A*, (+)-pinoresinol–forming DP, DRR206. Several residues lining the DRR206 active-site pocket are differentially conserved in (+)- or (–)-pinoresinol–forming DPs, suggesting a role in determining substrate orientation. The Ω loop is *labeled* to indicate proximity to the backside of the active site. *B*, PsPTS1, residue numbers differ by one (less) from those in GePTS1. *C*, modeled GhDIR4.

The (+)- and (–)-pinoresinol–forming DP (DRR206 and AtDIR6) structures contain an Ω loop that folds back upon the exterior of the barrel, with conserved residues (Thr^84^ and Ser^91^) that form a small cluster with His^39^ located on the barrel (Fig. 8*A*). This loop is also present in the medicarpin-forming (His^48/49^, Thr^85/86^, and Ser^92/93^ in PsPTS1/GePTS1, Fig. 8*B* and Fig. 6 (*C–E*)) and gossypol-forming (His^46^, Ser^88^, and Arg^81^, in place of Thr, in GhDIR4; Fig. 8*C*) DPs, and it appears to be a general feature of dirigent proteins. The conserved residues and structure of this loop and its position on the exterior of the barrel suggest that it may be either a locus of interaction with other proteins or that it may mediate flexibility in the upper portion of the barrel that comprises the active site.

The second exterior loop between β1 and β1′ in the medicarpin-forming DPs (Fig. 7*C*) is also similar to that found in the (–)-pinoresinol–forming DP AtDIR6 (Fig. 7*B*), but not in DRR206 (Fig. 7*A*), where an alternate transition directly to a larger, more disordered β1-β2 loop is found. It is conceivable that the disordered loop in DRR206 is capable of forming the additional β-strand and intervening loop. The proximity of the β1-β1' loop to the Ω loop is noteworthy. A conserved β-bulge found in both GePTS1 and PsPTS1 structures is also observed in AtDIR6, but not in DRR206, near the end of β7. The significance, if any, of these observations is currently unknown.

The medicarpin-forming DP (GePTS1 and PsPTS1) structures, being distant from both DRR206 and AtDIR6 in sequence space, also provided additional homology modeling leverage, particularly in the large DIR-b/d family. Their structures helped to clarify ambiguity in how sequence alignments of distantly related DPs might be constructed, particularly in β8, which has comparatively little sequence conservation throughout the DP superfamily (Fig. 3). Insofar as Tyr^181^ is on β8 in GePTS1 (Figs. 3 and 6*B*) (and Tyr^180^ in PsPTS1; Fig. 8*B*), it may be that additional functionally important residues in other subfamily classes of DPs are also located along this strand.

Our alignments also suggest that the β-strands forming the core β-barrel structures in all three DP types are largely conserved, this in turn indicating that homology modeling may be used to model and understand the active sites and, in particular, the surrounding loops. These are depicted in *dark blue* in the alignment (Fig. 3) and vary significantly in both length and sequence. We currently hypothesize that these loops hold important roles, possibly helping confer substrate specificity. Loops on the opposite end of the barrel (Fig. 7) are more conserved, particularly β2-β3 (Ω) and β6-β7 loops, and may represent potential interaction sites for, for example, a DP-specific (per)oxidase in a putative protein supramolecular complex.

### Putative active-site pocket

From domain-swapping experiments giving different coupling stereoselectivities, we provisionally identified key regions for substrate binding and coupling in the putative active site (9). This, with the X-ray data, led to the deduction that each (+)-pinoresinol–forming DP (DRR206) monomer in the trimer has a prominent deep pocket at one end of the barrel, surrounded by flexible loops. We proposed that this pocket, oriented toward the outside of the trimer and lined with hydrophobic residues, is provisionally the substrate-binding site for (+)-pinoresinol formation (Fig. 8*A*). The volume of the pocket is large enough that two monolignol-derived substrates could bind in a single pocket. Similar conclusions were drawn from structure determination of the (–)-pinoresinol-forming AtDIR6 (22).

The putative (+)-pinoresinol-forming DP (DRR206) active-site cavity is shallower and broader than that in the pterocarpan synthases, GePTS1 and PsPTS1, harboring dirigent-like domains. This presumably is indicative of differences in size and geometry of the putatively bound substrates and QM intermediates (*e.g.* mono- *versus* bis-QMs). Our homology model of GhDIR4 suggests that the binding site is larger and more accessible than those of PTS1, DRR206, or AtDIR6, partly because six fewer residues comprise loop VI and adjacent portions of strands β5 and β6 to accommodate two bulkier hemigossypol substrates.

Some residues forming the putative binding/active site in the interior of the barrel are conserved between DRR206 (Fig. 8*A*), AtDIR6, GePTS1 (not shown), PsPTS1 (Fig. 8*B*), and GhDIR4 (Fig. 8*C*). A notable exception is Tyr^181^/Tyr^180^ (in GePTS1/PsPTS1), this being a conserved residue in the majority of DIR-b/d subfamily sequences, although not that of GhDIR4. Indeed, the sequences similar to GePTS1 and PsPTS1 are most likely homologous pterocarpan synthases from other legumes (Fig. S6). Conversely, a corresponding tyrosine is neither conserved in the pinoresinol-forming DPs, DRR206 or AtDIR6, in the DIR-a subfamily nor found in the gossypol-forming DP in the DIR-b/d subfamily. This may make sense, insofar as the gossypol-forming DP mechanism might be more like that of pinoresinol-forming DPs (in which Tyr^181^ is absent) given the similarity in their putative prochiral QM radical substrates.

GePTS1 and PsPTS1 sequences lack a conserved aspartate as found in pinoresinol-forming DPs (Asp^134^ in DRR206, Asp^137^ in AtDIR6). The conserved Asp^134^/Asp^137^ residue was proposed to reprotonate one of the bis-QM carbonyl oxygens to facilitate nucleophilic addition by the C-9 OH at C-7′ to form one of the cyclic ether rings of pinoresinol (Fig. 2*A*). This aspartate may not be needed, or Tyr^181^/Tyr^180^ or a water molecule might fulfill this role. In place of this Asp, Asn^137^ is conserved in PTS1 and many DIR-b/d sequences (Fig. S6). The GhDIR4 sequence lacks either asparagine or aspartate at the equivalent position but has aspartate at the subsequent position. This is located in loop VI, which is considerably truncated in GhDIR4 and thus may have a function similar to that proposed in AtDIR6 (22). Finally, Arg^145^ is highly conserved in DPs, although not GhDIR4; this residue is nearby the aforementioned Asp/Asn in the active site. However, in GhDIR4, Arg^130^ is a few residues away in the sequence and nearby, in loop VI, in the homology model, and could fulfill the same role as Arg^145^.

### Biochemical mechanism considerations in the medicarpin-, pinoresinol-, and gossypol-forming DPs

The major difference between these three DP types is their distinct substrate versatilities, reflecting differences in substrate recognition and binding in their active-site pockets, as well as product outcome. All three DP types use substrates that initially had a free phenolic group functionality in their aromatic ring(s) and, in the case of hemigossypol, in both rings. The coniferyl alcohol and hemigossypol-derived substrate radicals also have very different aromatic group substitutions, and these need to be understood better from a substrate-binding requirement. With the need for an oxidase to generate the presumed free radical species, how the DP and the oxidase(s) interact for stereoselective coupling also needs to be determined.

Following one-electron oxidation of the phenolic OH groups in coniferyl alcohol and hemigossypol, prior to coupling, the stereoselectivity of the coupling reactions requires that the prochiral substrates be bound and oriented such that coupling only occurs at the specific regio-centers and not at other potential coupling sites. In the absence of the DPs, these substrates only produce free radical–derived racemic products through coupling, some of which are nonregiospecific.

This leads to the question as to what is being bound in the DP active site prior to coupling. One-electron oxidation of the C-4 phenolic group in coniferyl alcohol and at the equivalent position in hemigossypol would generate intermediates (QM radicals) with similar extended delocalization. These intermediates then stereoselectively couple to afford the corresponding chiral bis-QM intermediates. Their DP active sites can thus be envisaged as able to possibly bind both the various electron-delocalized intermediate (free radical) monomers and the corresponding chiral bis-QM intermediates. The monomer binding and orientation in the active sites, however, control the stereoselectivity outcomes. Subsequent intramolecular cyclization (C–O bond formation) and re-aromatization presumably occur in these DP active sites as well (12, 22).

In contrast, the medicarpin-forming DPs, with their dirigent-like domains, apparently process chiral substrates, with the *R* stereochemistry of the OH functionality at C-4 being favored over the *S*-configuration for C–O bond formation. These data thus suggest that the chirality of the 4-OH group is of considerable importance for preferentially undergoing dehydration to generate the presumed QM intermediate (or a functional equivalent) prior to C–O bond formation. However, the presence of the aromatic 7-OH group also appears to be essential, presumably enabling generation of the putative QM intermediate prior to ring closure to afford the pterocarpan skeleta.

### Proposed pterocarpan synthase mechanism

Uchida *et al.* (19) proposed that conversion of DMI to medicarpin catalyzed by PTS would likely have two or more reaction steps and proceed via a QM intermediate, possibly involving different conformational states of the enzyme. Determination of the structure of PTS, together with evaluation of active-site residue mutants, and substrate and intermediate docking simulations now allow the proposed mechanism to begin to be evaluated in greater detail and in context of the positions of conserved residues in the active site.

At a minimum, the mechanism would likely require an acidic residue that protonates the 4-OH of DMI to facilitate its departure as H_2_O, thereby producing what is formally a benzylic carbocation. Another residue or a bound water could then reversibly accept the phenolic 7-OH proton of the benzo-dihydropyran ring, affording the *para*-QM intermediate (Fig. 2*C*). The mechanism would also likely require stabilization of this intermediate and promotion of attack by the phenolic 2′-OH on the QM carbon (C-4) through (or simultaneous with) removal of the hydroxyl proton to form the new partially reduced furan ring of medicarpin.

The conserved polar residues in the active site of GePTS1—Asp^50^, Asp^83^, Tyr^103^, Asn^137^, Arg^145^, and Tyr^181^—are likely to facilitate this mechanism. Mutagenesis of four of these residues showed significant effects on activity (Asn^137^ and Arg^145^ were not targeted for mutagenesis).

To investigate whether Asp^50^ or Tyr^103^ had any effect on conversion of *cis*-DMI and *trans*-DMI substrates, both residues were individually replaced with alanine and phenylalanine, respectively (*i.e.* Asp^50^ → Ala and Tyr^103^ → Phe). As shown in Table 1, these two mutations resulted in massive reductions in catalytic turnover (*i.e.* down to 1 and 3% for the *cis*-DMI (3*R*,4*R*) substrate and to 1.6 and 7.8% with the *trans*-DMI (3*S*,4*R*) isomer, relative to WT GePTS1). Moreover, for the *cis*-DMI (3*R*,4*R*) substrate, the *K_m_* values were much higher for both mutants (*i.e. K_m_* values of 1,175 and 555 μm *versus* 145 μm for WT GePTS1), with the *V*_max_ for each mutant also greatly attenuated (220 and 306 *versus* 2,674 picokatals/μg of protein). In addition, when the *trans*-DMI (3*S*,4*R*) isomer was used, the *K_m_* value for D50A was only slightly attenuated (*i.e. K_m_* of 520 μm *versus* 680 μm for WT GePTS1), whereas for Y103F, it greatly increased to 3,320 μm. On the other hand, *V*_max_ values were reduced down to 9 and 265 picokatals/μg of protein, respectively, *versus* 712 picokatals/μg of protein for WT GePTS1.

In the proposed mechanism for pterocarpan (medicarpin) formation by PTS, the QM forms *after* the chiral substrate (DMI) binds. Thus, one might expect polar active-site residues that are not conserved in pinoresinol-forming DPs to fulfill this additional function. The residues fitting this description are, in GePTS1 (Fig. 6, *A–E*), Asp^83^ (conserved in all PTS sequences and present but not highly conserved in some other DP sequences) and Tyr^181^ (conserved in all PTS sequences and many other Dir-b/d sequences although not GhDIR4 and absent in other DP sequences). To investigate this possibility, GePTS1 mutants, D83A and Y181F, were also obtained, and the resulting proteins were purified.

Kinetic data (Table 1) established that the D83A and Y181F mutations also had significant deleterious effects on catalytic turnover. With the *cis*-DMI (3*R*,4*R*) substrate, catalytic turnover was reduced to 1.2 and 1.5%, relative to WT GePTS1, whereas for the *trans*-DMI (3*S*,4*R*) isomer, the reductions were down to 8.3 and 3.0% of WT GePTS1 activity. For the *cis*-DMI isomer, *K_m_* values increased to 1,300 and 825 μm *versus* 145 μm for WT GePTS1. *V*_max_ values for each were also greatly attenuated (283 and 233 *versus* 2,674 picokatals/μg of protein for WT GePTS1. With the corresponding *trans*-DMI, however, the *K_m_* value for D83A was only slightly attenuated (*i.e. K_m_* of 665 *versus* 680 μm for WT GePTS1), whereas for Y181F it was greatly increased to 5,665 μm. *V*_max_ determinations were also found to be attenuated (58 and 180 picokatals/μg of protein *versus* 712 picokatals/μg of protein for WT GePTS1). In other words, both of these mutations also overall had massive deleterious effects on catalytic turnover.

These effects on PTS1 activity from mutagenesis of Asp^50^, Asp^83^, Tyr^103^, and Tyr^181^, combined with the inferences from docked substrate and intermediate orientations, can thus be used to propose the following roles for polar active-site residues in the proposed mechanism.

In GePTS1, Tyr^181^ or a nearby bound water in the active site may have a role in accepting the 7-OH proton during formation of the QM intermediate, particularly if Tyr^181^ exists as the phenolate, which could be stabilized by the nearby side-chain of Arg^145^. Alternatively, Tyr^181^ and Arg^145^ may facilitate QM formation with a bound water as proton acceptor, rather than the phenolate directly. Tyr^181^ or a nearby protonated water would also presumably reprotonate the QM oxygen (7-O) upon cyclization at C-4 to form the new furan-like ring in medicarpin, regenerating the original (phenol) OH functionality. Docking predictions having DMI and DMI-QM structures, where the incipient QM is buried most deeply in the active site, suggest this role for Tyr^181^. Notably, Tyr^181^ is not conserved in pinoresinol-forming DPs, such as AtDIR6 and DRR206.

Asp^50^ is likely the donor that initially protonates the 4-OH, which then leaves as water to ultimately generate the QM. This same residue could then provide a negative charge to stabilize the partial positive charge on the QM carbon and could subsequently serve as a proton acceptor for the 2′-OH proton during attack on the QM carbon (C-4) through which cyclization to form the new partially reduced furan ring occurs. The proposed mechanism does, however, require that Asp^50^ be in an un-ionized form for the initial step, to protonate the 4-OH, and provisionally suggests that catalysis would be inhibited by low pH.

Docking experiments identified several bound DMI and DMI-QM orientations with the 7-OH directed inward toward the bottom of the active-site tunnel and near Arg^145^ and Tyr^181^. Among these orientations were some in which the 4-OH of DMI (see Fig. 6, *C–E*) and C-4 of DMI QM are proximal to Asp^50^. Asp^50^ in GePTS1 appears to form a hydrogen bond with Tyr^103^ and is also near Asp^83^; both residues are conserved in PTS1 sequences (Tyr^103^ is conserved widely across DPs) and may help modulate the proton donor and/or acceptor activity of Asp^50^.

Our working hypothesis for the role of Arg^145^, which is highly conserved in most dirigent proteins and dirigent-like domains, including the pinoresinol-forming dirigent proteins, is that the positively charged side-chain guanidino group stabilizes the QM intermediate by balancing the partial negative charge on the QM carbonyl oxygen. Whereas QMs are frequently drawn as a half-quinone (*e.g.* a 2,5-cycohexadienone with an exocyclic double bond to a benzylic carbon *para* to the carbonyl), it is useful to consider the zwitterionic resonance form: a phenolate with a benzylic carbocation at the *para* position. Stabilization of a reactive species with highly electron-rich and electron-poor moieties likely requires suitably located charged groups. In the proposed mechanism for PTS1, the likely role of the conserved arginine (Arg^145^ in GePTS1) is to stabilize the partial negative charge on the QM carbonyl oxygen, whereas the conserved aspartate (Asp^50^ in GePTS1) likely stabilizes the QM benzylic carbon and facilitates nucleophilic attack by a hydroxyl group to form a furan-like ring.

Asp^50^, Tyr^103^, and Arg^145^ are conserved in both AtDIR6 and DRR206 (Fig. 3), and the positions of the side-chains are nearly identical in the superposition of all three structures. This suggests a common role for these residues, despite the apparent dissimilarity in their substrates, including the likelihood that AtDIR6 and DRR206 bind two coniferyl alcohol QM radicals, whereas GePTS1/PsPTS1 bind a single DMI substrate and is unlikely to involve a QM radical in the mechanism. A *p*-QM has an electrophilic carbon at the benzylic position, *para* to a partially negatively charged carbonyl oxygen. Protonation of this oxygen decreases the energy barrier of the second cyclization step by making the benzylic carbon (C-4) considerably more electrophilic (31–33).

In GePTS1, the phenolic 2′-OH group, which attacks the electrophilic C-4 of the DMI-QM intermediate, is equivalent to either one of the nucleophilic oxygens (9- or 9′-OH) in the bis-QM intermediate en route to pinoresinol formation, which attack the electrophilic C-7′ and C-7 atoms, respectively, in this intermediate. In both substrates, the nucleophilic OH and electrophilic carbon are separated by three carbons, such that intramolecular cyclization forms a five-membered cyclic ether, a reduced furan (or partially reduced in the case of DMI). Both Asp^50^ in GePTS1 and its homologue in pinoresinol-forming DPs (Asp^49^ in AtDIR6) therefore presumably could have similar roles in stabilizing the partial positive charge on the QM carbon as well as in accepting the proton from the nucleophilic hydroxyl group.

In the pinoresinol-forming dirigent proteins DRR206 and AtDIR6, where the proposed mechanism has a bound bis-QM intermediate resulting from 8–8′ coupling of two coniferyl alcohol QM radicals, the homologous residue to Asp^50^ (Asp^49^ in AtDIR6) was proposed to have a somewhat different role. There it was envisaged as protonating the carbonyl oxygen at one end of the bis-QM, making the proximal methide carbon more electrophilic (formally resembling a benzylic carbocation) and facilitating cyclization in that half of the bis-QM, via attack by the nucleophilic 9-OH originating from the other coniferyl alcohol radical substrate, and thereby forming one of the cyclic ether rings in pinoresinol (22).

However, our interpretation is that, as suggested by the enzyme kinetics of GePTS1 (19) and data herein, there may be conformational change in the protein upon binding and/or rearrangement of the position of the substrate. We note that the barrel itself in GePTS1 and the conformations of residues inside it, particularly Phe^48^ and Asp^50^ (Fig. 3), are potentially influenced by His^49^ (on the outside of the barrel and in contact with conserved residues in the Ω loop). These influences may exert subtle effects on the active site either through interactions with a partner protein or in response to other stimuli.

Thus, perhaps significantly, Phe^48^, His^49^, and Asp^50^ in GePTS1 are conserved in nearly all dirigent proteins (Fig. 3) (whose equivalents in PsPTS1 are Phe^47^, His^48^, and Asp^49^; Fig. 8*B*). In addition, the role of conserved residue Arg^145^ (conserved in many DP sequences, including DRR206 and AtDIR6) is currently unproven, as discussed above, but a role in stabilization of the partial negative charge on the QM oxygen (at C-7) remains a reasonable assumption. Resolution of these and other ambiguities will likely require crystallization of PTS with bound substrates, products, intermediates, or their analogues.

## Concluding remarks

The key mechanistic aspects of the three DP types herein are (*a*) binding of monomeric species (achiral or chiral), (*b*) QM formation and binding (or a radical or ionic counterpart) (*e.g.* either via intermolecular coupling and bis-QM generation or mono-QM generation), and (*c*) re-aromatization (through either intramolecular cyclization (C–O bond formation) or intramolecular rearrangement).

It appears that in all three DP types (medicarpin-, pinoresinol-, and gossypol-forming DPs), the active site must be able to accommodate and stabilize QM intermediates. Assuming these are generated, both the lignan- and pterocarpan-forming DPs can then undergo intramolecular cyclization (C–O bond formation) to afford the corresponding products. However, whether this occurs at the DP active sites or following release of the mono- or bis-QM intermediates remains to be established. This differs, however, from the aromatic terpenoid (+)-gossypol–forming DP, which undergoes re-aromatization, with the latter occurring also either before or after release from the DP active site. The DP active sites thus can accommodate either at least two monomers for coupling or alternatively larger molecules for further processing (here intramolecular cyclization to afford pterocarpans).

These insights, we propose, will be of critical importance in both predicting and establishing the precise biochemical roles of the vast DP multigene families awaiting discovery in the future and in establishing the full diversity of the metabolic pathways involved, leading to different plant phenol metabolic classes. Clearly, any distinct land plant phenol metabolic class entry point (*e.g.* to lignans, lignins, aromatic diterpenoids, and pterocarpans thus far) requiring formation of QM intermediates (or a radical or ionic counterpart) can now be considered as having genes encoding either a DP or DP-like function.

In years gone by, terpenes were considered by some researchers to be produced nonenzymatically, but this notion evaporated when terpene synthases were discovered. As DP functions in land plant metabolism and evolution are identified, the importance of how such organisms actually control QM biochemistries will be perhaps key to better understanding how successful land plant adaptation originated and evolved.

## Experimental procedures

### Materials

All solvents and reagents were purchased from either Sigma–Aldrich or Fischer Scientific. Racemic vestitone was purchased from Santa Cruz Biotechnology, Inc., and synthetic (+)-medicarpin was kindly provided by Dr. K. H. Lee (University of North Carolina, Chapel Hill, NC, USA).

### Instrumentation and chromatography materials

High-resolution liquid chromatography electrospray ionization MS analysis of *cis-* and *trans*-DMI and medicarpin was performed in the negative ion mode using a Xevo G2 Qtof/ACQUITY Ultra Performance LC system (Waters) equipped with a BEH C_18_ column (Waters, 1.7-μm particles, 2.1 × 100 mm). Sodium formate (5 mm in 2-propanol-water (90:10, v/v)) was used for calibrating the mass spectrometer, and leucine enkephalin (2 ng/μl in acetonitrile-water containing 0.1% HCO_2_H (50:50, v/v)) was employed as lock-mass.

Chiral separations were carried out either on an Alliance 2690 HPLC system (Waters, Milford, MA, USA) with a photodiode array detector (model 2990, Waters) equipped with a Chiralcel OJ column (250 × 4.6 mm; Chiral Technologies, West Chester, PA, USA) eluted with hexanes-ethanol (7:3, v/v; flow rate, 0.3 ml/min) or on a Waters Acquity ultraperformance liquid chromatography system equipped with a chiral RU-2 column (150 × 4.6 mm; Shiseido, Tokyo, Japan) eluted with acetonitrile-water (6:4, v/v; flow rate, 0.2 ml/min). Detection was at 280 nm.

NMR spectra were recorded on a Varian VNMRS spectrometer operating at 599.64 and 150.79 MHz for ^1^H and ^13^C, respectively, and equipped with a 5-mm HCN cryoprobe (Varian) with a cold carbon preamp. The sample temperature was maintained at 20 °C for all experiments. *J* values are given in Hz. One-dimensional ^1^H and ^13^C and two-dimensional gHSQCAD, gHMBCAD, and gCOSY spectra (Figs. S8–S18) were acquired for both *cis*- and *trans*-DMI using typical acquisition and processing parameters. For the *cis*-DMI sample, a HOMO2DJ experiment was also acquired to aid in resolving the peak positions and *J*-coupling on a multiplet region centered at 6.39 ppm (see spectra in Figs. S8 and S10). Chemical shifts were referenced internally to the solvent methanol-*d*_4_ (3.31 ppm for the residual methyl proton and 49.15 ppm for C). For full NMR acquisition details, see the supporting information.

### Synthesis of cis-DMI ((3R,4R) and (3S,4S)) and trans-DMI ((3S,4R) and (3R,4S))

The four stereoisomers of *cis*- and *trans*-DMI were chemically prepared by sodium borohydride (NaBH_4_) reduction of racemic vestitone as described (19, 34). To a solution of racemic (3*RS*)-vestitone (40 mg) in ethanol (2 ml) was added NaBH_4_ (80 mg) at room temperature. The contents were stirred for 2–3 h until the vestitone was totally reduced to the corresponding DMI. After completion of the reaction, excess ethanol was removed *in vacuo* and the reaction mixture was quenched with water (3 ml), and the whole was extracted with ethyl acetate (2 × 30 ml). The ethyl acetate solubles were combined and passed through an anhydrous Na_2_SO_4_ plug and evaporated to dryness *in vacuo*. The residue so obtained was subjected to silica gel preparative TLC as described in Uchida *et al*. (19) using toluene/ethyl acetate/methanol/benzene (6:4:1:3) to individually afford *cis*-DMI ((3*R*,4*R*) and (3*S*,4*S*)) (11.7 mg) and *trans*-DMI ((3*S*,4*R*) and (3*R*,4*S*)) (21.5 mg), respectively.

*cis*-DMI ((3*R*,4*R*) and (3*S*,4*S*)): δ_H_ (methanol-*d*_4_, 600 MHz) 3.49 (1 H, td, *J* 3.2, 12.4, H-3), 3.73 (3 H, s, 4′-OMe), 4.11 (1 H, ddd, *J* 1.4, 3.6, 10.2, H-2a), 4.52 (1 H, dd, *J* 10.2, 12.5, H-2b), 4.72 (1 H, dd, *J* 1.3, 3.1, H-4), 6.26 (1 H, d, *J* 2.4, H-8), 6.37 (dd, *J* 2.5, 8.3, H-6), 6.39 (1 H, dd, *J* 2.5, 8.3, H-5′), 6.41 (1 H, d, *J* 2.1, H-3′), 6.98 (1 H, d, *J* 8.2, H-6′), 7.07 (1 H, d, *J* 8.3, H-5) (Fig. S8). δ_C_ (methanol-*d*_4_, 151 MHz) 39.5 (C-3), 55.7 (4′-OMe), 65.4 (C-2), 66.7 (C-4), 102.5 (C-3′), 103.7 (C-8), 105.7 (C-5′), 109.4 (C-6), 118.0 (C-4a), 118.8 (C-1′), 130.6 (C-6′), 132.9 (C-5), 156.7 (C-8a), 157.5 (C-2′), 159.8 (C-7), 161.2 (C-4′) (Fig. S9). *m/z* = 287.0927 [M–H]^–^ (calc. mass for C_16_H_15_O_5_ 287.0919).

*trans*-DMI ((3*S*,4*R*) and (3*R*,4*S*)): δ_H_ (methanol-*d*_4_, 600 MHz) 3.39 (1 H, td, *J* 3.5, 6.2 Hz, H-3), 3.70 (3 H, s, 4′-OMe), 4.24 (1 H, dd, *J* 6.4, 11.0, H-2a), 4.30 (1 H, dd, *J* 3.6, 10.9, H-2b), 4.84 (1 H, d, *J* 6.0, H-4), 6.21 (1 H, d, *J* 2.4, H-8), 6.29 (1 H, dd, *J* 2.6, 8.5, H-5′), 6.38 (1 H, d, *J* 2.7, H-3′), 6.40 (1 H, dd, *J* 2.4, 8.7, H-6), 6.91 (1 H, d, *J* 8.5, H-6′), 7.19 (1 H, d, *J* 8.4, H-5) (Fig. S14). δ_C_ (methanol-*d*_4_, 151 MHz) 41.6 (C-3), 55.7 (4′-OMe), 67.9 (C-2), 68.2 (C-4), 102.5 (C-3′), 103.5 (C-8), 105.6 (C-5′), 109.7 (C-6), 117.9 (C-4a), 119.6 (C-1′), 129.8 (C-6′), 131.6 (C-5), 157.1 (C-8a), 157.6 (C-2′), 159.3 (C-7), 161.1 (C-4′) (Fig. S15). *m/z* = 287.0920 [M–H]^–^ (calc. mass for C_16_H_15_O_5_ 287.0919).

### Cloning and heterologous expression of G. echinata pterocarpan synthase 1 (GePTS1) and mutants

*GePTS1* coding sequence (GenBank™ accession no. LC121822), as well as four individual mutants (D50A, D83A, Y103F, and Y181F) were codon-optimized for *E. coli* and synthesized via GeneOptimizer^®^ (Invitrogen) without the N-terminal signal peptide (23 amino acids). The *GePTS1* gene was cloned into the pET101/D-TOPO^®^ *E. coli* expression vector, whereas the four mutants were cloned into the pET100/D-TOPO^®^. GePTS1 and each of the four mutant constructs were transformed into One Shot^®^ BL21 Star™ (DE3) competent *E. coli* (Invitrogen) according to the manufacturer's protocol. Initial Luria-Bertani medium cultures (10 ml) containing 100 μg/ml carbenicillin were incubated overnight (∼15 h) at 37 °C with shaking at 250 rpm. A 500-μl aliquot of each culture was then used to inoculate Luria-Bertani medium (50 ml) containing 100 μg/ml carbenicillin. After incubating at 37 °C with shaking at 250 rpm to obtain an *A*_600_ of ∼0.6, the cultures were induced with isopropyl 1-thio-β-d-galactopyranoside at a final concentration of 1 mm. After continued shaking at 28 °C for 24 h, cells were harvested by centrifugation at 3,000 × *g* for 20 min at 4 °C, with the pellets frozen and stored at –80 °C.

### Cloning and heterologous expression of P. sativum medicarpin–forming DP (PsPTS1)

The *GePTS1* sequence was used to search the *P. sativum* “Cam_eor” UniGene set (24), resulting in a gene (PsCam039127) being selected and named *PsPTS1*. PsPTS1 had ∼92%/85% similarity/identity to the GePTS1 peptide sequence. The *PsPTS1* coding sequence was codon-optimized for *E. coli* and synthesized as above without its N-terminal signal peptide (21 amino acids). Cloning and expression of *PsPTS1* were performed using the *GePTS1* protocols above.

### Purification of GePTS1, PsPTS1, and the four GePTS1 mutant His tag fusion proteins

Pelleted cultures were individually lysed using BugBuster^®^ Protein Extraction Reagent (EMD Millipore) with Benzonase^®^ Nuclease and rLysozyme™ added. Purification of each protein was individually performed using a POROS™ 20 MC metal chelate affinity (Thermo Scientific) column. Each cell-free extract was applied to the POROS™ 20 MC column equilibrated in binding buffer (20 mm Tris-HCl, pH 7.9, 500 mm NaCl, and 20 mm imidazole) at 4 °C and then washed with 10 bed volumes of binding buffer to remove unbound proteins. Each recombinant protein was next eluted using elution buffer (20 mm Tris-HCl, pH 7.9, 500 mm NaCl) containing imidazole initially at a concentration of 150 mm and then 300 mm.

Individual fractions of each recombinant proteins were subjected to SDS-PAGE using a Mini-PROTEAN^®^ TGX™ precast gel, 4–20% gradient (Bio-Rad), with visualization done by silver staining. Fractions containing each of the recombinant proteins (Fig. S1) were individually pooled, and the buffer was exchanged to 25 mm Tris-HCl (pH 7.9) using a PD10 column (GE Healthcare), following which the resulting protein solutions were individually concentrated using an Amicon^®^ Ultra-4 10K centrifugal filter (Millipore). Protein quantification was carried out using the Bradford assay (Bio-Rad) microassay procedure. Typically, 5–7 mg of each pure protein were obtained from a 30-ml *E. coli* culture.

### Gel-permeation chromatography

GePTS1 (10.5 μg, 5 μl) and PsPTS1 (13.5 μg, 5 μl) were individually loaded onto a TSKgel G3000SW_XL_ column (300 × 7.8 mm, 10-μm particle size; Tosoh Bioscience) equilibrated in 0.1 m NaH_2_PO_4_-Na_2_HPO_4_ buffer (pH 7.0) containing NaCl (0.3 M) at a flow rate of 0.2 ml/min. Molecular weights were estimated by comparison of their elution profiles with protein standards, thyroglobulin (669,000), apoferritin (443,000), β-amylase (200,000), alcohol dehydrogenase (150,000), BSA (66,000), and carbonic anhydrase (29,000), respectively. Blue dextran was used to determine void volume.

### CD spectrophotometry

CD spectra of WT GePTS1 and mutants were recorded on an AVIV model 410 CD spectrophotometer. Samples were dissolved in 20 mm Tris-HCl buffer, pH 7.9. Protein concentration ranged from 170 to 650 μg/ml and was measured as above. Spectra were recorded at 25 °C in 1-mm quartz cuvettes over a wavelength range from 270 to 190–200 nm, depending on concentration. Data were collected with 0.5-nm wavelength steps, 1.0-nm bandwidth, and 1.0-s averaging time. Four scans were averaged, and a buffer blank was collected prior to each sample and subtracted from the average of the four scans. Spectra were not smoothed but were normalized to the same concentration for comparability.

### Enzyme assays for analysis of substrate enantiomer depletion

Enzyme assays with either purified recombinant GePTS1, PsPTS1, or GePTS1 mutants were performed as described (19). Assay mixtures consisted of 0.1 m NaH_2_PO_4_-Na_2_HPO_4_ buffer (pH 6.6, 220 μl), *cis*-DMI ((3*R*,4*R*) and (3*S*,4*S*)), or *trans*-DMI ((3*S*,4*R*) and (3*R*,4*S*)) (41 μm, 10 μl), BSA (0.1 μg/μl, 10 μl), and the purified DP (10 μl). After incubation at 30 °C for 30 min, each assay was extracted with EtOAc (2 × 0.5 ml), dried *in vacuo*, and resuspended in EtOH (40 μl), with an aliquot (5 μl) subjected to chiral HPLC analysis on a Chiralcel OJ column (see “Instrumentation and chromatography materials” and Fig. S2).

### Kinetic parameter determination

To determine the kinetic parameters of all purified recombinant PTSs, assays were performed as described above, using 10 different substrate concentrations (100–500 μm) and carried out in triplicates. Incubations were for 5 min with the following protein concentrations for *cis*- and *trans*-DMI as substrate: GePTS1 (10 and 80 ng), PsPTS1 (50 and 300 ng), D50A (500 ng and 20 μg), D83A (100 and 600 ng), Y103F (100 and 800 ng), and Y181F (100 ng and 1.6 μg). After incubation, each assay was extracted with EtOAc (2 × 0.5 ml), dried *in vacuo*, and resuspended in MeOH–H_2_O (7:3, v/v; 30 μl), with an aliquot (5 μl) subjected to chiral HPLC analysis on a RU-2 column (see “Instrumentation and chromatography materials”).

### Crystallization and X-ray data collection

Initial crystallization conditions for GePTS1 and PsPTS1 were obtained using the microbatch-under-oil method employing 1,536-well microassay plate high-throughput screening (25) at the Hauptman Woodward Institute (Buffalo, NY). Microcrystals were obtained under 43 conditions: conditions 168, 243, 296, 510, 655, 778, and 934 (for GePTS1) and conditions 13, 100, 258, 315, 320, 340, 369, 406, 503, 514, 540, 941, 950, 957, 1,001, and 1,258 (for PsPTS1) were preliminarily selected to be scaled up in-house as hanging-drop vapor diffusion methods on VDX 24-well plates (Hampton Research). These were incubated at 21 °C with a drop size of 3 μl consisting of equal volumes of reservoir solution and protein at a concentration of 11.8 and 7.6 mg/ml for GePTS1 and PsPTS1, respectively, in 25 mm Tris-HCl buffer (pH 7.9). Each drop was equilibrated against a 500-μl reservoir.

For GePTS1, two conditions from those initial hits produced crystals: condition 243, 0.1 m ammonium chloride, 0.1 m sodium citrate, pH 4.2, 12% PEG 2000 (w/v); condition 296, 0.1 m sodium phosphate, 0.1 m sodium citrate, pH 4.2, 12% PEG 20000 (w/v). Condition 296 was further optimized, and diffraction quality crystals were obtained with 0.1 m sodium phosphate-monobasic, 0.18 m sodium citrate, pH 4.2, 9% PEG 20000 (w/v).

For PsPTS1, five conditions from the initial hits produced crystals: condition 13, 0.5 m sodium acetate trihydrate, pH 4.9, 15% (w/v) PEG 3350; condition 100, 5% (w/v) ethyl ammonium nitrate, 0.09 m MES, pH 5.8, 27% (w/v) PEG 3350; condition 315, 0.1 m sodium ammonium chloride, 0.1 m MES, pH 6, 24% (w/v) PEG 20000; condition 340, 0.1 m potassium bromide, 0.1 m MES, pH 6, 24% (w/v) PEG 20000; condition 1258, 0.2 m ammonium acetate, 0.1 m sodium acetate trihydrate, pH 4.6, 30% (v/v) PEG 4000. Condition 13 was further optimized, and diffraction quality crystals were obtained with 0.25 m sodium acetate trihydrate, pH 4.9, 10% (w/v) PEG 3350.

GePTS1 and PsPTS1 crystals were subsequently flash-cooled in crystallization buffer, supplemented with either 25% (v/v) glycerol or ethylene glycol in H_2_O, stored in cryo-vials, and shipped to the Stanford Synchrotron Radiation Light source (SSRL) for data collection. The GePTS1 crystals belong to the trigonal space group P3_1_21 with unit cell dimensions *a* = *b* = 162.572, *c* = 99.763, diffracted to ∼2.6 Å resolution. A complete data set comprising 1,000 images with a rotation angle of 0.2° was collected from a single crystal on SSRL beamline BL9-2 using X-rays at 12,658 eV (0.97946 Å) and a PILATUS 6M PAD detector running in the shutterless mode. Data were processed with XDS (35) and scaled with AIMLESS (36) from the CCP4 suite of programs (37). The Matthews coefficient (38), assuming six molecules in the asymmetric unit, was 3.1 Å^3^/Da (60% solvent content). Final data collection statistics are given in Table 2 (39–42). The PsPTS1 crystals belong to the cubic space group P2_1_3 with unit cell dimensions *a* = *b* = *c* = 78.893, diffracting to ∼1.5 Å resolution. A complete data set comprising 900 images with a rotation angle of 0.2° was collected from a single crystal on SSRL beamline BL9-2 using X-rays at 12,658 eV (0.97946 Å) and a PILATUS 6M PAD detector running in the shutterless mode. Data were processed with XDS (35) and scaled with AIMLESS (36). The Matthews coefficient (38), assuming one molecule in the asymmetric unit, was 2.53 Å^3^/Da (51% solvent content). Final data collection statistics are given in Table 2. An additional data set from a second cryo-cooled crystal was collected on BL9-2 using X-rays at 7,500 eV (1.65307 Å) via the inverse beam method and wedges of 30° to maximize the anomalous signal from the intrinsic sulfur atoms. A complete data set comprising 1,800 0.2° images was collected and also processed with XDS (35) and scaled with AIMLESS (36). Statistics are given in Table 2.

**Table 2:** **Data collection statistics** Numbers in parentheses relate to the highest-resolution shell (2.69–2.60 Å for GePTS1, 1.51–1.48 Å for PsPTS1, and 1.89–1.85 Å for PsPST1 sulfur-SAD).

	GePTS1	PsPTS1	PsPTS1 sufur-SAD
Space group	P3_1_21	P2_1_3	P2_1_3
Resolution range (Å)	39.1–2.60	35.3–1.48	39.4–1.85
Reflections (observed/unique)	539,183/46,983	209,128/27,529	517,060/14,218
*R*_meas_*^a^* (%)	0.125 (1.821)	0.073 (1.065)	0.079 (1.228)
*R*_pim_*^b^* (%)	0.037 (0.526)	0.026 (0.387)	0.013 (0.255)
I/σ	13.3 (1.7)	14.6 (1.8)	28.4 (2.8)
Completeness (%)	100.0 (100.0)	99.9 (98.8)	100 (99.6)
CC_½_*^c^*	0.997 (0.598)	0.999 (0.72)	1.000 (0.834)
Multiplicity	11.5 (11.7)	7.6 (7.4)	36.4 (22.4)
Wilson *B* (Å^2^)	80.4	18.8	36.1
Anomalous resolution limit (Å)			3.3
Anomalous completeness			100 (99.3)
Anomalous multiplicity			19.1 (11.5)
CC_anom_*^d^*			0.197

*^a^ R*_meas_ is the redundancy-independent merging *R* factor (39).

*^b^ R*_pim_ is the precision-indicating merging *R* factor (40).

*^c^* Percentage of correlation between intensities from random half-sets of data (41).

*^d^* Correlation of Δ*I*_anom_ from two random half-sets (42).

### Data processing, structure determination, and refinement

The GePTS1 structure was solved by molecular replacement using a starting model derived from the dirigent protein AtDIR6 from *A. thaliana* (PDB code 5LAL) (22). Two models were used comprising (*a*) monomer AtDIR6 and (*b*) trimeric AtDIR6. GePTS1 and AtDIR6 sequences were aligned, and both AtDIR6 models were converted into pseudo-GePTS1 models using the program CHAINSAW (43) from the CCP4 suite (37), whereby identical residues in the two sequences were retained, and those that differed were truncated at the Cβ atom. A good molecular replacement solution was obtained using the trimeric pseudo-GePTS1 model (searching for two copies) using the program PHASER in the PHENIX suite (44), with a translation function Z-score (TFZ) of 47.2 and a log-likelihood gain (LLG) after refinement of 2,432. The same solution was obtained using the monomeric pseudo-GePTS1 model (TFZ = 34.5, LLG = 2442), searching for six copies. This latter solution was submitted to a round of automated model building with PHENIX.AUTOBUILD using data to 2.65 Å resolution, giving a crystallographic *R*_work_ and *R*_free_ of 0.313 and 0.367, respectively, with 824 residues built in 43 fragments covering the six molecules in the asymmetric unit. Refinement of the GePTS1 structure using all data to 2.6 Å resolution was completed with PHENIX.REFINE (44), alternating with manual building of the model using the molecular graphics program COOT (28). Water molecules were added at structurally and chemically relevant positions, and the atomic displacement parameters for all atoms in the structure were refined isotropically. Final refinement statistics are given in Table 3 (45).

**Table 3:** **Structure refinement statistics**

	GePTS1	PsPTS1
PDB code	6OOC	6OOD
Resolution range (Å)	39.0–2.6	35.3–1.50
*R*-factor/*R*_free_*^a^*	0.1969/0.2385	0.1624/0.1907
Reflections used, total/free		26,219/1,315
**Total atoms**		
Protein	7,813	1,240
Solvent	101	186
***B* factors**		
Protein (Å^2^)	66.9	20.6
Solvent (Å^2^)	60.5	33.3
**RMSD from ideality**		
Bonds (Å)	0.009	0.005
1-3 distances (Å)	1.09	0.892
**Ramachandran plot**		
Residues in preferred regions*^b^* (%)	92.1	96.7
Outliers	18	0
Molprobity score	2.05*^c^*	1.41*^d^*

*^a^ R*_free_ was calculated with 5% of the reflections.

*^b^* As defined in MOLPROBITY (45).

*^c^* In the 95th percentile of structures at similar resolution.

*^d^* In the 83rd percentile of structures at similar resolution.

The PsPTS1 structure was solved by sulfur-SAD methods implemented in PHENIX. Following solvent flattening and density modification, the overall figure of merit was 0.325. Autobuilding in PHENIX generated a model comprising 95 of 169 expected residues. Initial refinement with PHENIX.REFINE gave *R*_work_ and *R*_free_ values of 0.33 and 0.35, respectively. The model was rebuilt into the density-modified electron density, and subsequent refinement was switched to the 1.5 Å resolution native data. Water molecules were added at structurally and chemically relevant positions, with atomic displacement parameters for all atoms in the structure refined isotropically. Final refinement statistics are given in Table 3. Final coordinates and structure factors have been deposited in the Protein Data Bank with accession codes 6OOC (GePTS1) and 6OOD (PsPTS1).

### Substrate docking

Ligand-protein docking simulations were set up, run, and analyzed in the Windows version of AutoDockTools (ADT version 1.5.6), a graphical user interface to the AutoDock 4 suite of programs for predicting binding of small molecules (substrates, inhibitors) to a macromolecular receptor's 3D structure (46). The four substrate (DMI) diastereomers and two QM intermediate enantiomers, with two alternate conformations of the flavone pyran ring where the 3′ phenol substituent was either pseudo-equatorial or pseudo-axial for each (12 structures total), were built and energy-minimized in Chem3D and saved in .mol2 format. Docking simulations were performed with AutoGrid and AutoDock. AutoGrid was used to precompute the grid maps of interaction energies for various atom types in the ligand with the enzyme. These grid maps were then used in the AutoDock docking simulations to determine the total ligand-protein interaction energy. To prepare the structures of the ligand and the protein for docking, missing hydrogen atoms and Gasteiger partial atomic charges were added to their 3D structures loaded from their respective .mol2 and .pdb files. The water molecules present with the enzyme structure were removed. AutoDockTools identified five active torsions in the ligand. The grid box was centered upon the enzyme with a grid spacing of 0.375 Å, with sufficient size to cover the ligand- and the receptor-binding sites. No motion was permitted in the protein backbone or side chains. After the structures were prepared, AutoGrid was run to obtain the grid maps for the AutoDock calculations. The AutoDock calculations were executed using the Lamarckian genetic algorithm with 100 dockings per ligand and 2,500,000 energy evaluations per docking. Finally, 100 enzyme-bound ligand conformations were obtained and analyzed.

### Phylogenetic tree construction

Sequences annotated as dirigent protein were obtained from UniProt for *A. thaliana*, cotton (*G. hirsutum*), grape (*Vitis vinifera*), *Sorghum bicolor*, aspen (*Populus tremuloides*), castor bean (*Ricinus communis*), barley (*Hordeum vulgare*), soybean (*Glycine max*), *Medicago truncatula*, *Brachypodium distachyon*, rice (*Oryza*, several species), maize (*Zea mays*), Sitka spruce (*Picea sitchensis*), loblolly pine (*Pinus taeda*), *S. moellendorffii*, and *P. patens*. Truncated sequences lacking sequence alignment coverage of all eight β-strands were culled, duplicate sequences were removed, and very long leader/trailer peptides and very large loop insertions were trimmed. A multiple-sequence alignment was built with Clustal Omega (EBI), and the tree was rendered with iTOL (47) as an unrooted tree (Fig. 1).

## Data availability

Coordinates and structure factors for GePTS1 and PsPTS1 have been deposited in the Protein Data Bank with accession codes 6OOC and 6OOD, respectively. All other data are contained within the article and the supporting information.

## Supplementary Material

Supporting Information

## Footnotes

4 H. Van Etten, personal communication.
