# Supplementary material for: Pterocarpan synthase (PTS) structures suggest a common quinone methide–stabilizing function in dirigent proteins and proteins with dirigent-like domains
Source: J Biol Chem. 2020 Jun 21;295(33):11584–601. doi: 10.1074/jbc.RA120.012444 (PMC7450108; doi:10.1074/jbc.RA120.012444)
Supplement: Supporting Information [file supp_295_33_11584__index.html]

Pterocarpan synthase (PTS) structures suggest a common quinone methide–stabilizing function in dirigent proteins and proteins with dirigent-like domains — Pterocarpan synthases and dirigent-like protein domains — Supporting Information 

# Pterocarpan synthase (PTS) structures suggest a common quinone methide–stabilizing function in dirigent proteins and proteins with dirigent-like domains

## Supporting Information

- Supporting Information (to be published online) - Supplementary Methods, Table and Figures
