# Supplementary material for: Pterocarpan synthase (PTS) structures suggest a common quinone methide–stabilizing function in dirigent proteins and proteins with dirigent-like domains
Source: J Biol Chem. 2020 Jun 21;295(33):11584–601. doi: 10.1074/jbc.RA120.012444 (PMC7450108; doi:10.1074/jbc.RA120.012444)
Supplement: Supporting Information [file supp_RA120.012444_157926_2_supp_550148_qc35bs.pdf]

Supporting Information

NMR experimental parameters

NMR spectra were recorded on a Varian VNMRS spectrometer operating at 599.64 and 150.79 MHz for ^{1}H and ^{13}C, respectively, and equipped with a 5 mm HCN cryoprobe (Varian) with cold carbon preamp and a z-axis gradient. The sample temperature was maintained at 20 °C for all experiments. Chemical shifts were referenced internally to the solvent metahnol-d_4 (3.31ppm for the residual methyl proton and 49.15 ppm for C). 1D ^{1}H spectra having a spectral width of 11.1 ppm were acquired with 8 transients each using a full 90° pulse, acquisition time of 4 s (27k complex points zero-filled to 128k), and relaxation delay of 10 s. ^{13}C 1D spectra were acquired with a 250 ppm spectral width using 45° flip-angle pulses, 0.87 s acquisition time (32k complex points, zero-filled to 128k points), and 2 s relaxation delay. Acquisition of 1024 (*trans*-DMI) or 2048 (*cis*-DMI) transients co-added for each spectrum was preceded by 8 steady-state scans, broadband, power-gated ^{1}H decoupling was accomplished using the WALTZ-16 composite pulse decoupling scheme, and 1 Hz line-broadening was applied during processing. All 2D experiments had 32 steady-state scans applied prior to acquisition and incorporated default forward linear prediction during processing. 2D multiplicity-edited gHSQCAD experiments had spectral widths of 11.0 and 120.0 ppm in the direct and indirect dimensions, respectively, and were acquired with 16 transients (acquisition time of 0.15 s, 1036 complex points), with a relaxation delay of 1.2 s, for each of 196 increments comprising the indirect dimension. A one-bond $^{1}J_{CH}$ of 146 Hz was used. Broadband ^{13}C decoupling using the WURST composite pulse decoupling scheme was active during acquisition. Both dimensions were zero-filled to a final size of 2,048 points and subjected to a Gaussian apodization function. The gHMBCAD experiments had spectral widths of 16.0 and 160.0 ppm in the direct and indirect dimensions respectively and were acquired with 32 transients (acquisition time of 0.15 s, 1,442 points), with a 1.2 s relaxation delay, for each of the 200 increments acquired in the indirect dimension. A multiple-bond coupling constant of 8 Hz was used as well as one-bond suppression for $^{1}J_{CH}$ in the 130 – 165 Hz range. Each dimension was zero-filled to 2,048 points, sine bell and Gaussian apodization were applied in the direct and indirect dimensions respectively. The gCOSY spectra were acquired with a 10.0 ppm spectral width in both dimensions, only 1 transient (acquisition time of 0.3 s, 1,803 points) was acquired for each of 256 increments in the indirect dimension with a relaxation delay of 1 s. Post-acquisition processing included zero-filling to 2,048 points and sine bell apodization in both dimensions. For the cis-DMI sample, a HOMO2DJ experiment was also acquired to aid in resolving the peak positions and J-coupling on a multiplet region centered at 6.39 ppm (see spectra in Figures S8 and S10). The HOMO2DJ experiment consisted of spectral widths of 16.0 ppm and 64 Hz in the direct and indirect dimensions respectively and was acquired with 4 transients per each of the 80 indirect dimension increments with an acquisition time of 1.25 s (12,019 complex points) and using a relaxation delay of 2 s. Post-acquisition processing included zero-filling to a final data size of 16,384 and 512 complex points in the direct and indirect dimensions, application of a sine bell apodization function in both dimensions and, finally, subjected to a 45° tilt and was symmetrized.

Table S1

Structural superpositions[a]

	PsPTS1	GePTS1	DRR206	AtDIR6
PsPTS1	–	0.6 (152 core Cα)	1.6 (111 core Cα)	1.4 (135 core Cα)
GePTS1	0.6 (456 core Cα)<br>1.5 (all Cα)	–	1.5 (113 core Cα)	1.4 (135 core Cα)
DRR206	1.7 (335 core Cα)<br>9.0 (all Cα)	1.7 (341 core Cα)<br>10.2 (all Cα)	–	1.7 (117 core Cα)
AtDIR6	1.5 (408 core Cα)<br>4.0 (all Cα)	1.5 (404 core Cα)<br>7.1 (all Cα)	1.8 (251 core Cα),<br>6.6 (all Cα)	–

[a] The upper sector shows the superpositions of individual monomers. Values given are averages over all the molecules in the asymmetric unit. The lower section pertains to the trimeric structure superpositions. The *rmsd* values are in Å and the number of Cα positions is given in parentheses for the matched core atoms from SSM, or for all Cα atoms using ICM-Pro.

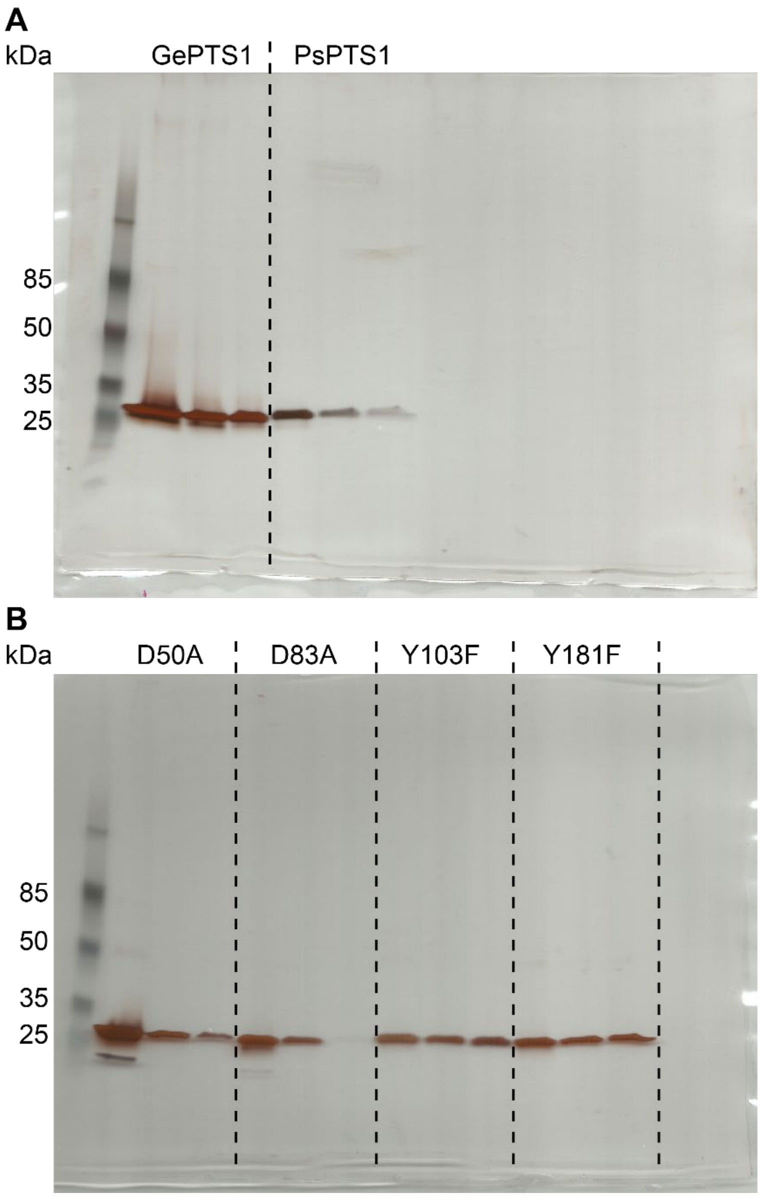

Figure S1. Purification of PTSs. *A,* GePTS1 and PsPTS1. *B*, GePTS1 mutants, D50A, D83A, Y103F and Y181F. The three lanes for each protein show elution with 300 mM imidazole (3 × 500 μl).

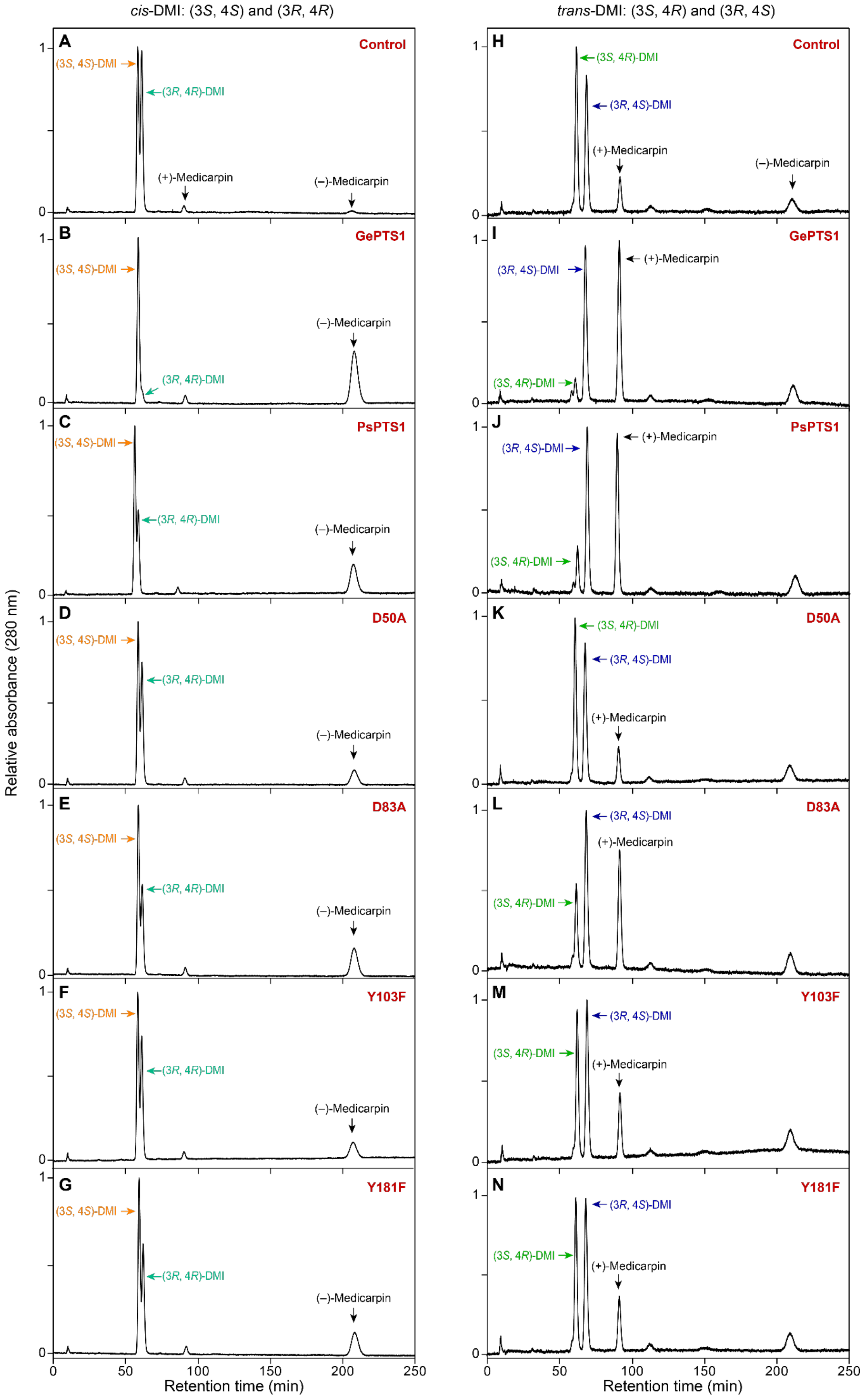
cis-DMI: (3S, 4S) and (3R, 4R)
trans-DMI: (3S, 4R) and (3R, 4S)
A Control
B GePTS1
C PsPTS1
D D50A
E D83A
F Y103F
G Y181F
H Control
I GePTS1
J PsPTS1
K D50A
L D83A
M Y103F
N Y181F
(3S, 4S)-DMI
(3R, 4R)-DMI
(3S, 4R)-DMI
(3R, 4S)-DMI
(+)-Medicarpin
(−)-Medicarpin
Relative absorbance (280 nm)
Retention time (min)

Figure S2. Medicarpin formation from *cis*-DMI ((3*R*,4*R*) and (3*S*,4*S*)) and *trans*-DMI ((3*S*,4*R*) and (3*R*,4*S*)). *A* and *H*, control (no dirigent protein) with *cis*-DMI ((3*R*,4*R*) and (3*S*,4*S*)) and *trans*-DMI ((3*S*,4*R*) and (3*R*,4*S*)), respectively. Following a 30 minute incubation, both (+)- and (–)-medicarpins formed non-enzymatically in small amount. *B* – *G*, incubation of *cis*-DMI ((3*R*,4*R*) and (3*S*,4*S*)) with GePTS1 (10 ng; *B*), PsPTS1 (10 ng; *C*), and GePTS1 mutants: D50A (1 µg; *D*), D83A (100 ng; *E*), Y103F (100 ng; *F*) and Y181F (100 ng; *G*). Following 30 minute incubation, depletion of (3*R*,4*R*)-DMI and formation of (–)-medicarpin is observed in each case. *I* – *N*, incubation of *trans*-DMI ((3*S*,4*R*) and (3*R*,4*S*)) with GePTS1 (500 ng; *I*), PsPTS1 (1 µg; *J*), and GePTS1 mutants: D50A (1 µg; *K*), D83A (1 µg; *L*), Y103F (1 µg; *M*) and Y181F (500 ng; *N*). Following 30 minute incubation, depletion of (3*S*,4*R*)-DMI and formation of (+)-medicarpin is observed in each case.

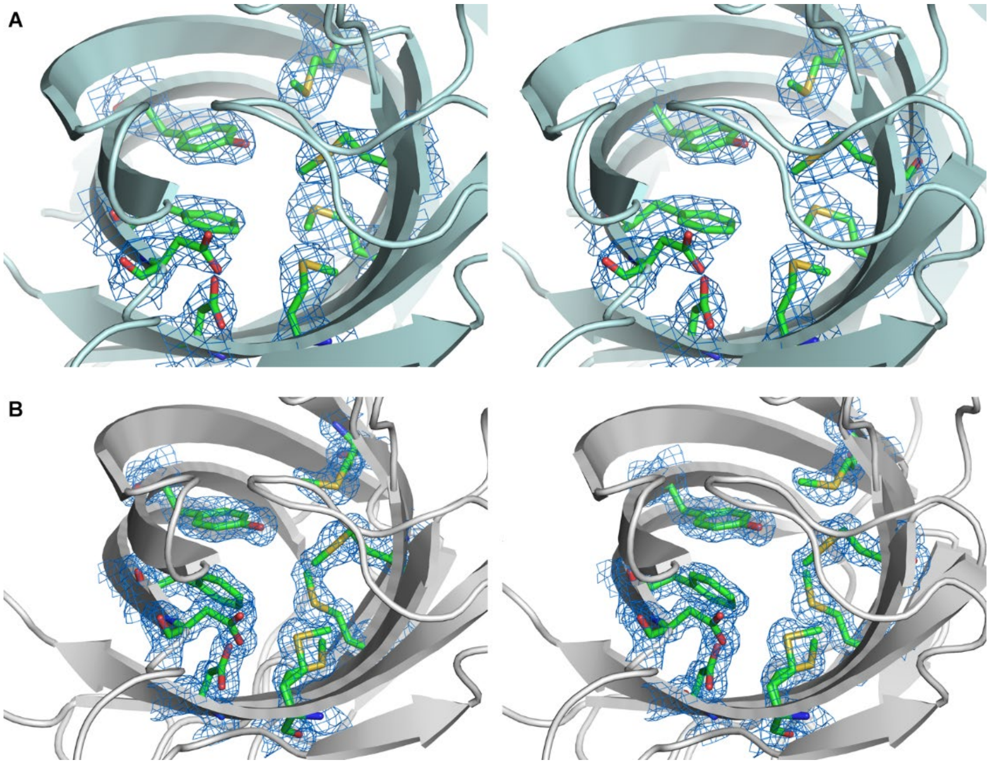

Figure S3. Electron density map. Final $2F_o$-F_c electron density (blue mesh, contoured at 1σ) for (*A*) GePTS1 and (*B*) PsPTS1 showing part of the map near the putative substrate binding site.

MTYYQSMSPTVLGFQEEKFTHLHFYFHDVVTGPKPSMVIVA
EPNGKAKNSLPFGTVVAMDDPLTVGPESDSKLVGKAQGIYT
SISQEEMGLMMVMTMAFSDGEFNGSTLSILARNMIMSEPVR
EMAIVGGTGAFRFARGYAQAKFYSVDFTKGDAIVEYDIFVF
HY**KGELNSKLEGKPIPNPLLGLDSTRTGHHHHHH**

Figure S4. GePTS1 sequence. The C-terminus His-Tag linker is in bold red.

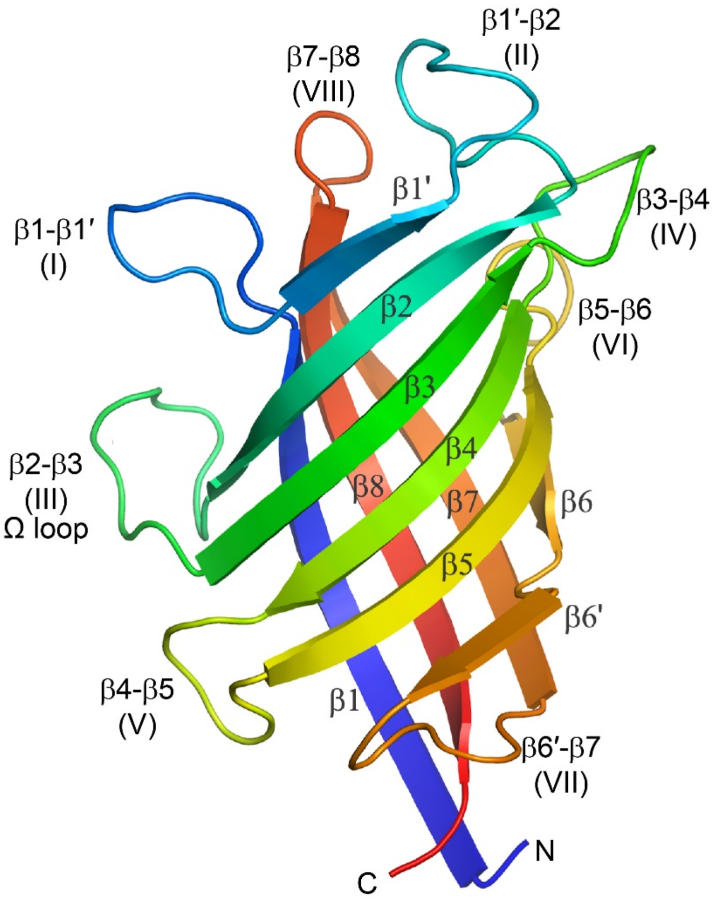

Figure S5. Structure of the PsPTS1 monomers. Ribbon representation of PsPTS1 rainbow colored from the N-terminus (blue) to the C-terminus (red). The β-strands are labeled the same as for the GePTS1 monomer (Fig. 5). The Ω-loop is indicated, and the loops surrounding the active site opening are at the top of the barrel.

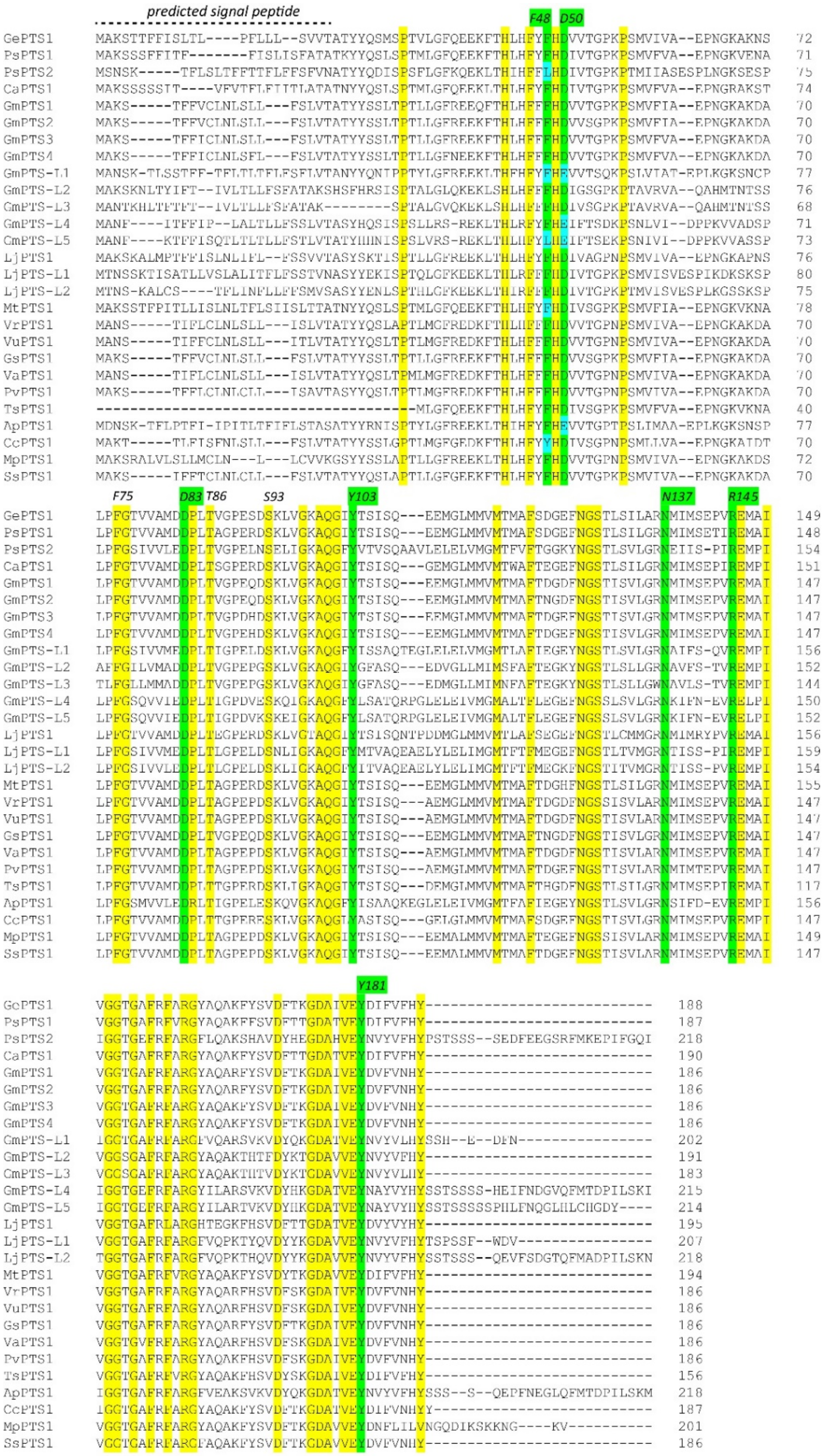
predicted signal peptide
F48
D50
F75
D83
T86
S93
Y103
N137
R145
Y181

Figure S6. Amino acid sequence alignment of PTS from Leguminosae. Alignment was created using Clustal Omega. ApPTS1 (Indian licorice, *Abrus precatorius*, XP_027352715.1), CaPTS1 (*Cicer arietinum*, XP_004493624.1), CcPTS1 (chickpea , *Cajanus cajan*, XP_020213585.1), GePTS1 (*Glycyrrhiza echinata*), (GmPTS1 (soybean, *Glycine max*, NP_001236934), GmPTS2 (GR830455), GmPTS3 (XP_003554228.1), GmPTS4 (NP_001353981.1), GmPTS-L1 (XP_003521234.1), GmPTS-L2 (XP_003554226.1), GmPTS-L3 (XP_003554227.1), GmPTS-L4 (NP_001236116.2), GmPTS-L5 (NP_001236254.2), GsPTS1 (*Glycine soja*, XP_028225437.1), LjPTS1 (*Lotus japonica*, FS322418), LjPTS-L1 (AFK47764.1), LjPTS-L2 (AFK41387.1), MpPTS1 (*Mucuna pruriens*, RDX58397.1), MtPTS1 (*Medicago truncatula*, XP_003625317.1), PsPTS1 and PsPTS2 (*Pisum sativum*), PvPTS1 (*Phaseolus vulgaris*, XP_007162362.1), SsPTS1 (*Spatholobus suberectus*, TKY62509.1), TsPTS1 (*Trifolium subterraneum*, GAU31514.1), VaPTS1 (adzuki bean, *Vigna angularis*, XP_017417783.1), VrPTS1 (mung bean, *Vigna radiata* var. *radiata*, XP_014495568.1), VuPTS1 (cowpea, *Vigna unguiculata*, XP_027939702.1).

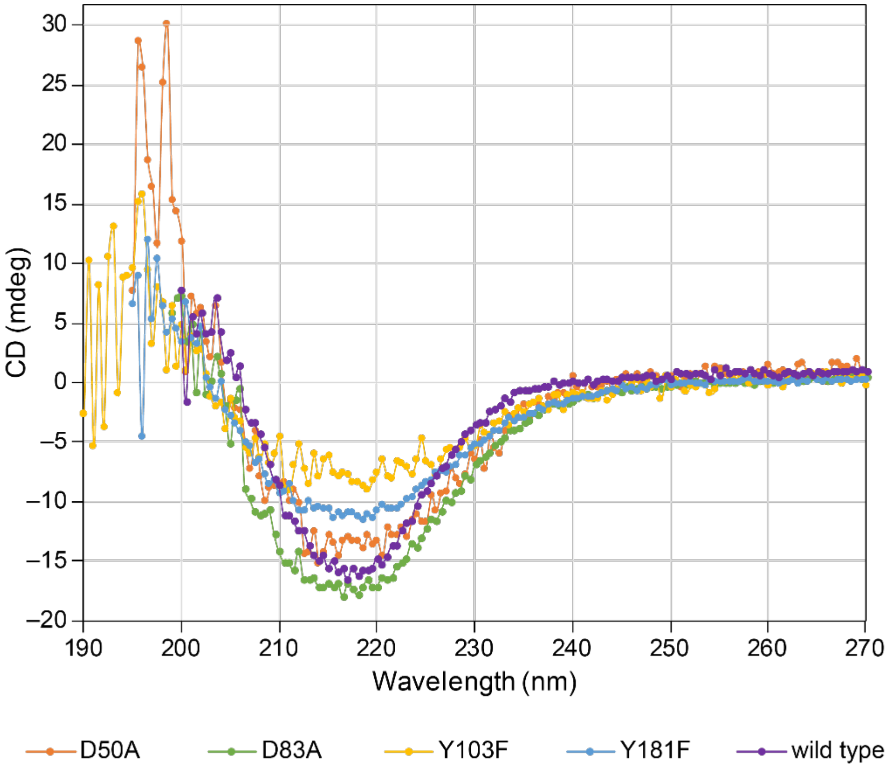

Figure S7. Circular dichroism spectra of GePTS1 wild type and corresponding mutants in 20 mM Tris-HCl, pH 7.9, 25 °C, normalized to reported concentrations

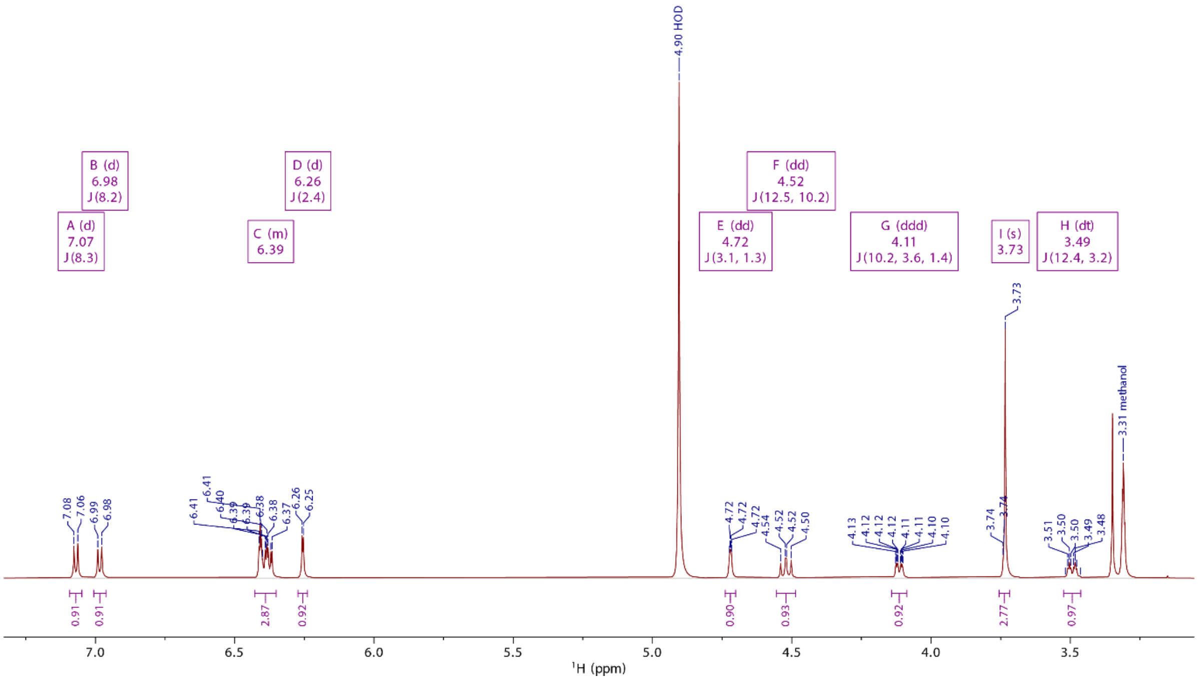

Figure S8. 1D ^{1}H NMR spectrum of *cis*-DMI in methanol- d_4 showing primary product multiplet analysis performed with MestreNova 14.0.1 (Mestrelab Research, S.L.). See Figure S10 for J-resolved spectrum of the multiplet at 6.39 ppm.

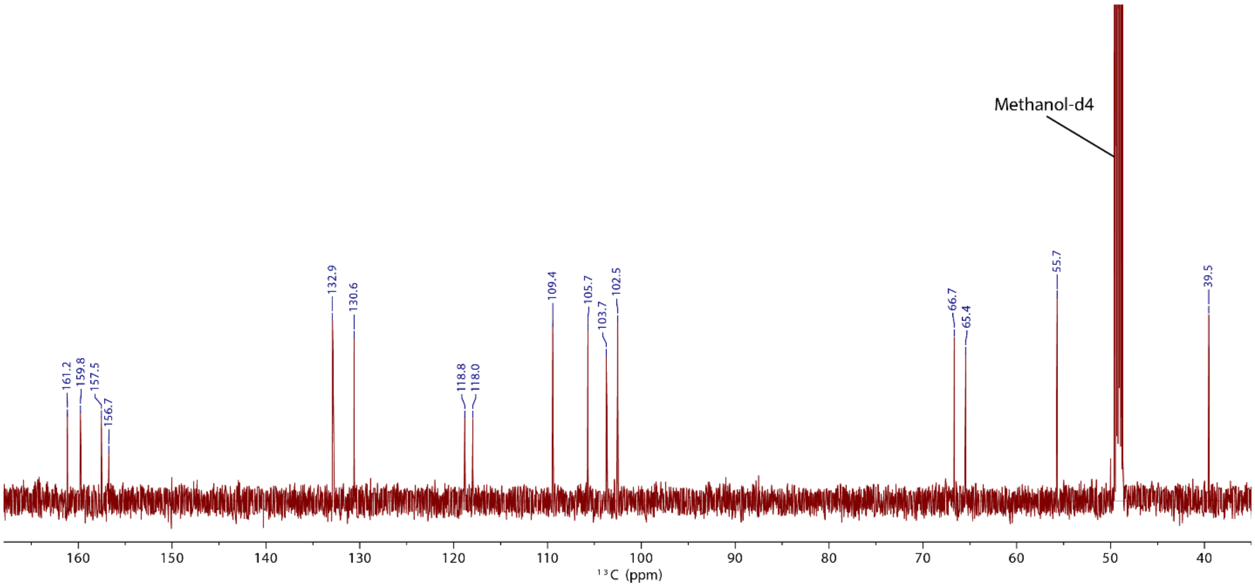

Figure S9 1D ^{13}C NMR spectrum of *cis*-DMI in methanol-d_4.

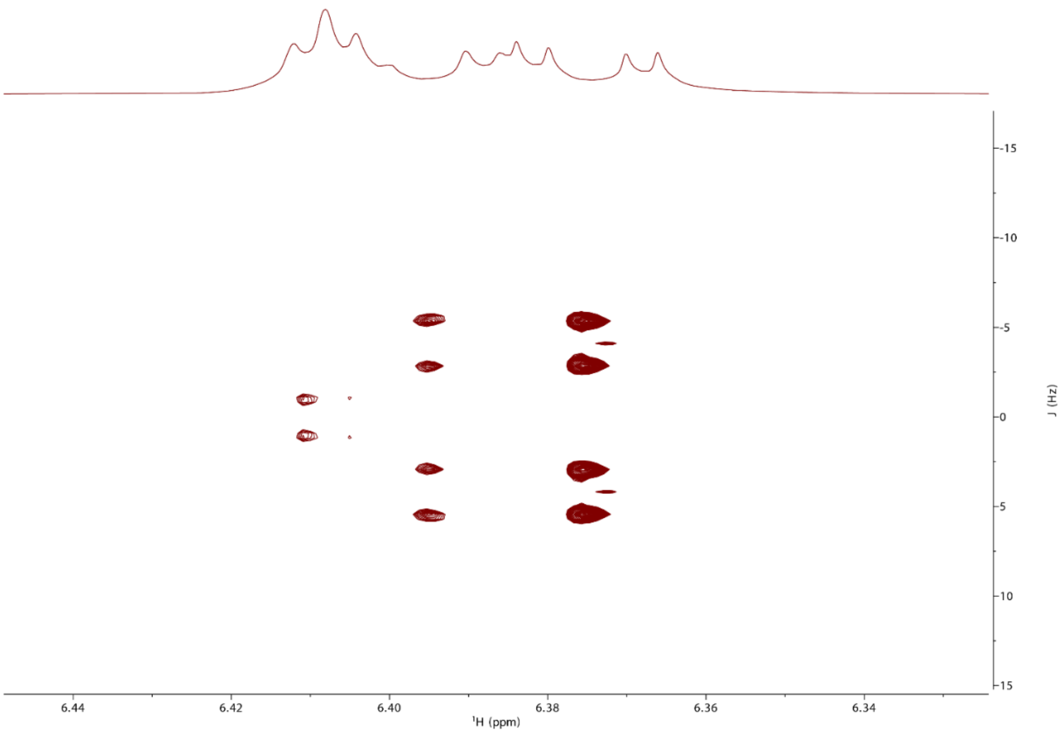

Figure S10. Portion of the 2D ^{1}H HOMO2DJ NMR spectrum of *cis*-DMI showing the overlapped multiplet at 6.39 ppm is comprised of peaks at 6.37 ppm (1 H, dd, J = 2.5 and 8.3 Hz), 6.39 ppm (1 H, dd, J = 2.5 and 8.5 Hz), and 6.41 ppm (d, J = 2.1 Hz).

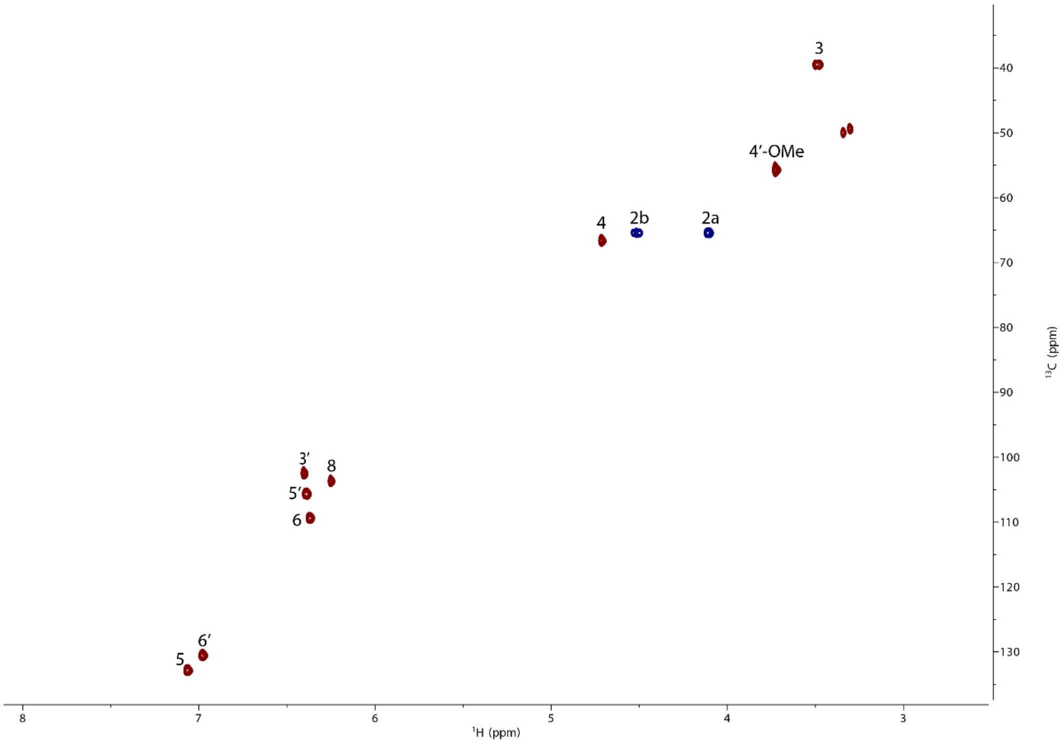

Figure S11. 2D multiplicity edited [CH, CH_3 up (red), CH_2 down (blue)] gHSQCAD NMR spectrum of *cis*-DMI annotated with assignments.

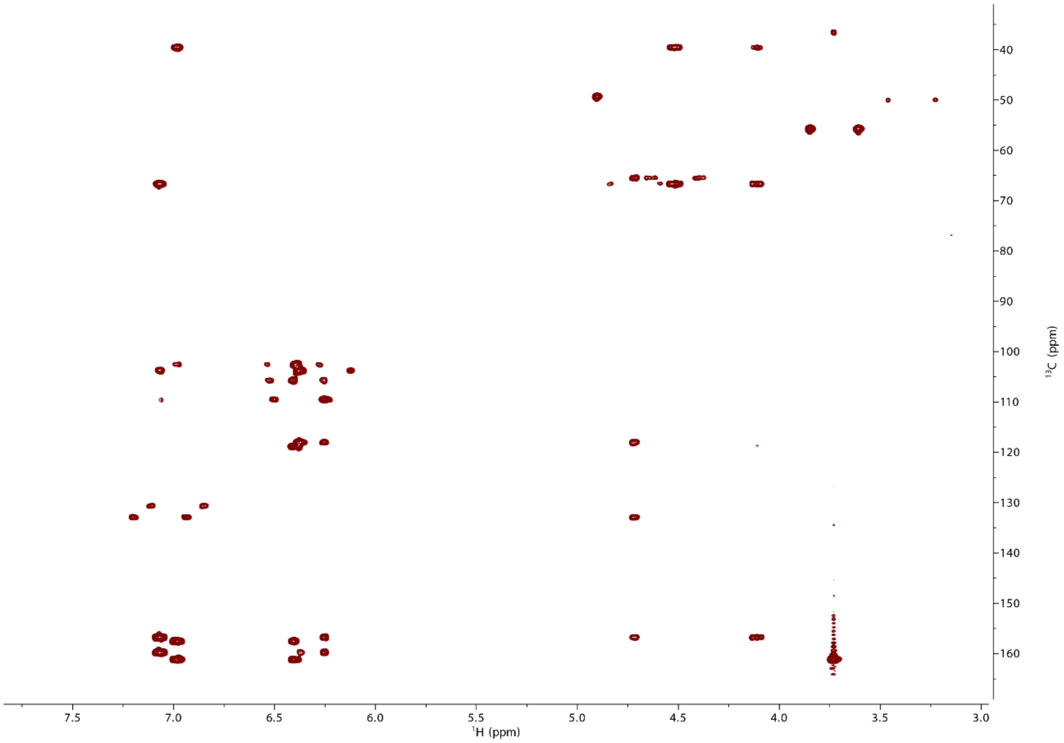

Figure S12. 2D gHMBCAD NMR spectrum of *cis*-DMI.

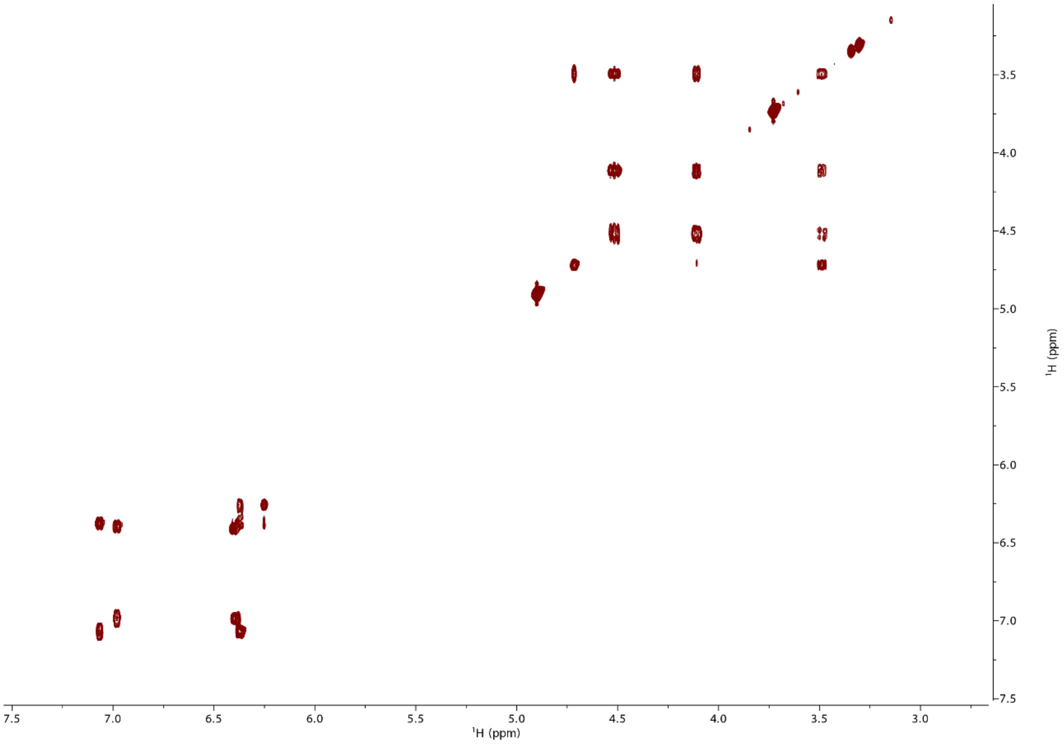

Figure S13. 2D gCOSY NMR spectrum of *cis*-DMI

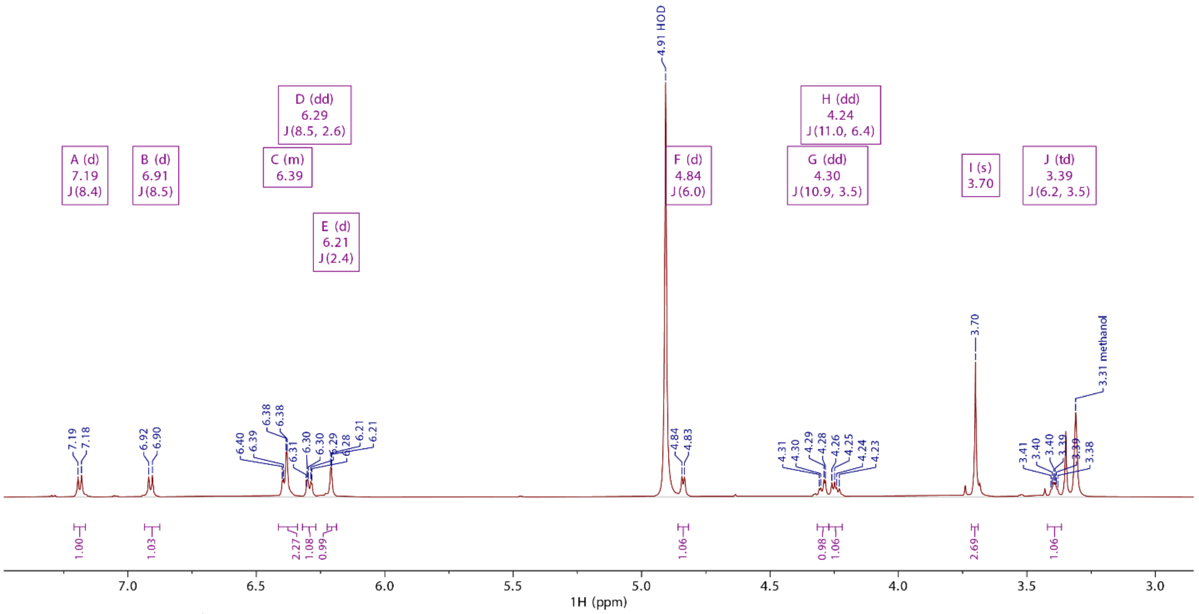

Figure S14. 1D ^{1}H NMR spectrum of *trans*-DMI in methanol-d_4 showing primary product multiplet analysis performed in MestreNova 14.0.1 (Mestrelab Research, S.L.). The multiplet centered at 6.39 ppm consists of peaks at 6.40 (1H, dd, J = 2.4, 8.7 Hz) and 6.38 (1H, d, J = 2.7 Hz) ppm.

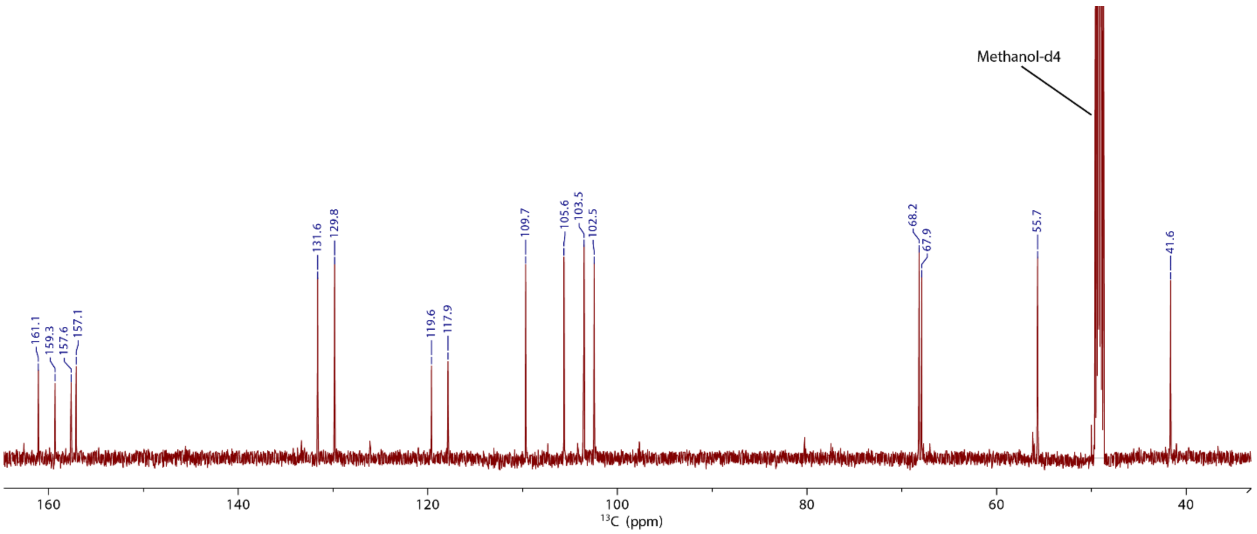

Figure S15. 1D ^{13}C NMR spectrum of *trans*-DMI in methanol-d_4.

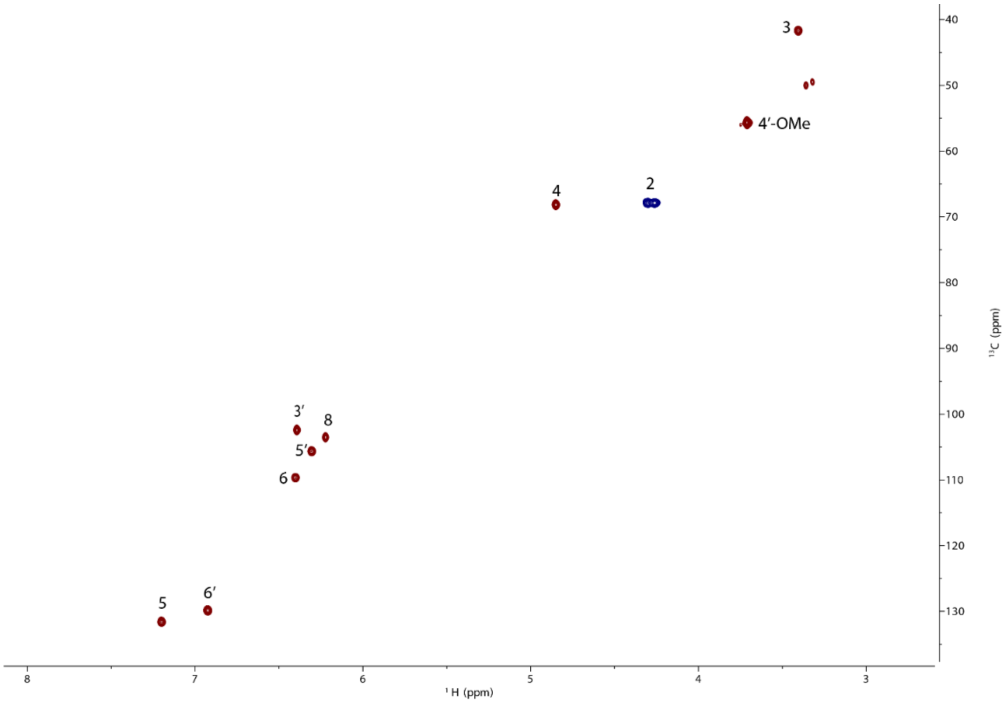

Figure S16. 2D multiplicity-edited [CH, CH_3 up (red), CH_2 down (blue)] gHSQCAD NMR spectrum of *trans*-DMI annotated with assignments.

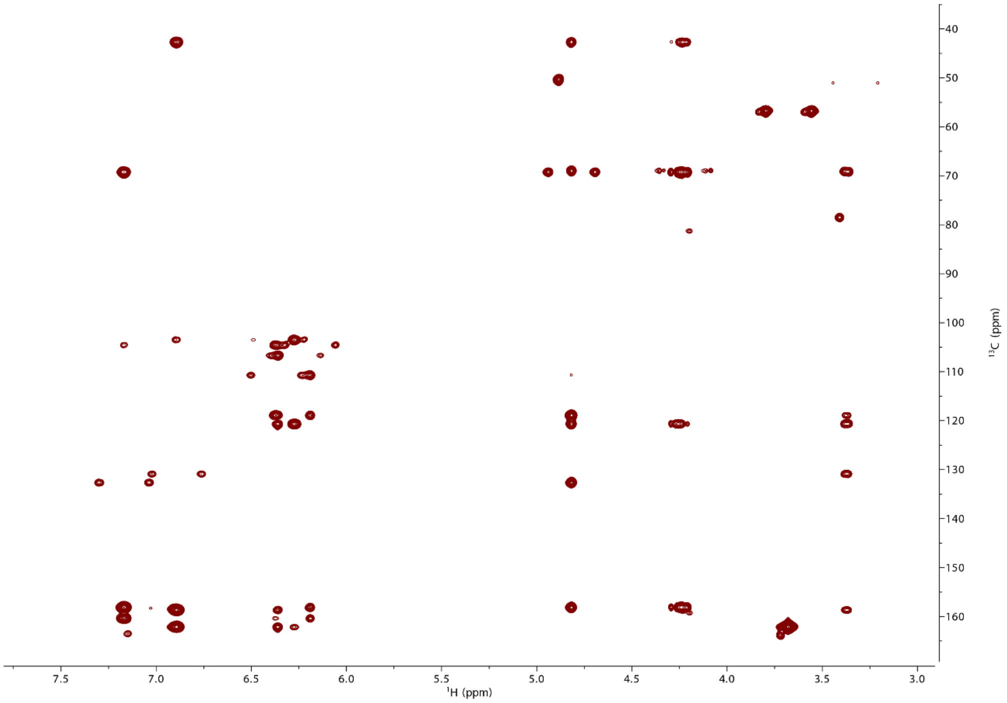

Figure S17. 2D gHMBCAD NMR spectrum of *trans*-DMI.

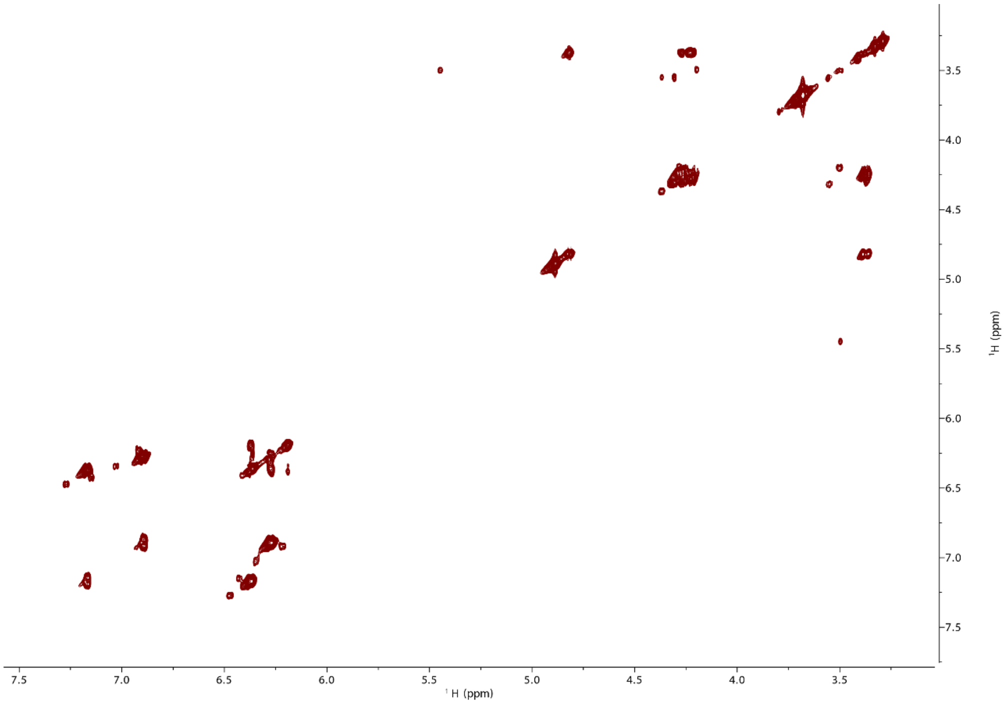

Figure S18. 2D gCOSY NMR spectrum of *trans*-DMI.
